# Highly Diverse *Phytophthora infestans* Populations Infecting Potato Crops in Pskov Region, North-West Russia

**DOI:** 10.3390/jof8050472

**Published:** 2022-04-30

**Authors:** Eve Runno-Paurson, Collins A. Agho, Nadezda Zoteyeva, Mati Koppel, Merili Hansen, Tiit Hallikma, David E. L. Cooke, Helina Nassar, Ülo Niinemets

**Affiliations:** 1 Institute of Agricultural and Environmental Sciences, Estonian University of Life Sciences, Kreutzwaldi 1, 51006 Tartu, Estonia; collinsaimuanvbosa@gmail.com (C.A.A.); mati.koppel@emu.ee (M.K.); merili_h@hotmail.com (M.H.); thallikma@gmail.com (T.H.); helina.nassar@emu.ee (H.N.); ylo.niinemets@emu.ee (Ü.N.); 2 N. I. Vavilov All-Russian Institute of Plant Genetic Resources, 190000 St. Petersburg, Russia; nzoteyeva@gmail.com; 3 The James Hutton Institute, Invergowrie, Dundee DD2 5DA, UK; david.cooke@hutton.ac.uk

**Keywords:** late blight, SSR markers, genetic diversity, mating type, metalaxyl resistance

## Abstract

There is limited understanding of the genetic variability in *Phytophthora infestans* in the major potato cultivation region of north-western Russia, where potato is grown primarily by small households with limited chemical treatment of late blight. In this study, the mating type, sensitivity to metalaxyl, and genotype and population genetic diversity (based on 12 simple sequence repeat (SSR) markers) of 238 isolates of *P. infestans* from the Pskov region during the years 2010–2013 were characterized. The aim was to examine the population structure, phenotypic and genotypic diversity, and the prevalent reproductive mode of *P. infestans*, as well as the influence of the location, time, and agricultural management practices on the pathogen population. The frequency of the A2 mating was stable over the four seasons and ranged from 33 to 48% of the sampled population. Both mating types occurred simultaneously in 90% of studied fields, suggesting the presence of sexual reproduction and oospore production in *P. infestans* in the Pskov region. Metalaxyl-sensitive isolates prevailed in all four years (72%), however, significantly fewer sensitive isolates were found in samples from large-scale conventional fields. A total of 50 alleles were detected in the 141 *P. infestans* isolates analyzed for genetic diversity. Amongst the 83 SSR multilocus genotypes (MLGs) detected, 65% were unique and the number of MLGs varied between locations from 3 to 20. These results, together with the high genotypic diversity observed in all the locations and the lack of significance of linkage disequilibrium, suggest that sexual recombination is likely responsible for the unique MLGs and the high genetic diversity found in the Pskov region population, resembling those of north-eastern European populations.

## 1. Introduction

The late blight pathogen, *Phytophthora infestans,* is a long studied pathogen that has been claimed to be the most destructive agent for potato production worldwide. This pathogen was responsible for the Irish famine in the mid-1840s and is a re-emerging disease that still constitutes a major threat to potato cultivation [1]. The cost of *P. infestans* infections due to yield losses and disease control of the pathogen has been estimated at around €1 billion in Europe alone [2]. This hemibiotrophic oomycete is heterothallic with two mating types (A1 and A2), which enable the pathogen to reproduce sexually and asexually [3].

Until 1980, only A1 mating type isolates were known to occur in Europe and other parts of the world, which has prevented the sexual reproduction of *P. infestans* across many countries, except in Mexico which was considered the center of origin where the A1 and A2 mating types co-existed [3]. However, the A2 mating type was introduced to Europe in shipments of potatoes from Mexico [4], and first collected in Europe in 1980 in eastern Germany [5], then in 1981 in Switzerland [6], England [7], the Netherlands [8], and Russia in the Leningrad region five years later in 1985 [9]. The presence of both mating types leads to sexual recombination and the production of oospores that can survive outside the host plant in the soil over a long period, even in dry and cold conditions [10]. The presence of sexual recombination increases the genetic diversity of the pathogen and also acts as an additional source of primary inoculum. This has resulted in drastic changes in the genetic structure of the populations of *P. infestans* and enhanced the spread and severity of the disease [4]. The increased genetic variability of the pathogen can lead to a more rapid rate of pathogen evolution and adaptation to different environmental conditions and new potato genotypes selected for their improved resistance to *P. infestans* [11]. The enhanced adaptability of the genetically more diverse *P. infestans* constitutes a good example of adaptation for survival with resultant difficulties in the management of the disease.

In the study period of 2010–2013, Russia ranked third in the world’s potato production, after China and India [12]. In Russia, the average annual potato consumption per person is about 120–130 kg [13], and the country is largely self-sufficient in potato production. The majority of potatoes in Russia are produced traditionally by small households for local consumption [14]. Small households accounted for 79% of potato production in 2012, whereas only 13% was produced by agricultural enterprises and 8% by private farms [15]. The share of potato production by small households has been estimated even greater, 93% by Elansky et al. [16] and 85% by Elansky et al. [17]. Due to the lack of late blight control, private plots and home gardens may serve as an important source of late blight infection [18]. Even in commercial potato fields, late blight control is insufficient, often resulting in the infection of seed material [17]. In Russia, the average yield losses caused by *P. infestans* in the period 2002–2017 were 33.6%, with the largest being in 2013 with a 66% loss [19].

Increased genotypic diversity in *P. infestans* populations is dependent on hybridization during the sexual process, the intensive exchange of low-quality infected seed materials, and the dispersal of sporangia in the atmosphere [17]. Rapid changes in the *P. infestans* population’s genetic structure increase the pathogen’s epidemiological potential [20]. Thus, the monitoring *P. infestans*’ population characteristics and genotypic structure is needed to gain an insight into the migration, evolution, and virulence of the pathogen, allowing there to be updates to management advice for potato cultivation and disease protection [13]. Long-term phenotypic studies (2000–2017) in one of the main potato growing regions in the Moscow region showed a very complex and diverse population of *P. infestans* with an increase of the frequency of the A2 mating type over time [21,22]. The genetic diversity of Russian populations of *P. infestans* has been analyzed based on SSR using a limited set of data from potato fields (65 isolates) for the period 2008–2011 in five regions: the Kostroma, Leningrad, Moscow, Nizhnii Novgorod, and Smolensk regions, where the highest diversity was in the potato population in the Moscow region and similarities with other European populations were not identified [13]. A more recent study with a few samples collected in 2013–2014 from Pushkin potato fields (Leningrad region) showed that the majority of isolates from 2014 resembled several variants of the common European genotypes EU_2_A1 and EU_4_A1 [23]. The use of disease-resistant potato cultivars is an integral part of sustainable and long-term management strategies for late blight. However, developing late-blight-resistant cultivars in the Pskov region, that has a human population of over 636,000 and potato cropping on an area of 7400 ha [24], is challenging because populations of *P. infestans* have never been studied in this part of north-western Russia, that borders the intensive large-scale potato cultivation areas in the northern Baltics. Such information is important for identifying suitable sources of resistance and pathogen isolates for use in resistance screening in potato breeding programs.

Potato late blight has considerable importance in Russian potato production. As described above, the populations of *P. infestans* in the European part of Russia and neighboring countries, such as Estonia and Latvia [25,26,27], are highly diverse, where disease control is difficult to implement, and thus yield losses are unavoidable. However, there is currently no information on the *P. infestans* populations in the Pskov region of Russia. Therefore, in this four year study, *P. infestans* isolates were collected from ten locations in the Pskov region in Russia and characterized with 12 SSR markers [28] and phenotypically with the mating type and resistance to metalaxyl to determine the genotypes, and the genetic and phenotypic diversity of this population. This is the first detailed survey of the situation of this particular region.

More specifically, it was necessary to find out: (1) how genetically diverse the *P. infestans* populations are in the Pskov region, as well as the diversity level caused by the sexually recombined strains; (2) what the phenotypic variation is within the Pskov population of *P. infestans*, indicated by the mating type and metalaxyl resistance; and (3) how the location, year, and agricultural management practices influence the genotypic diversity of the pathogen. Briefly, we also tried to compare the Pskov region’s *P. infestans* population with other Russian and European populations. We hypothesized that the *P. infestans* populations in the region are sexually and genetically diverse.

## 2. Materials and Methods

### 2.1. Collection and Isolation of P. infestans Strains

**Figure 1:**
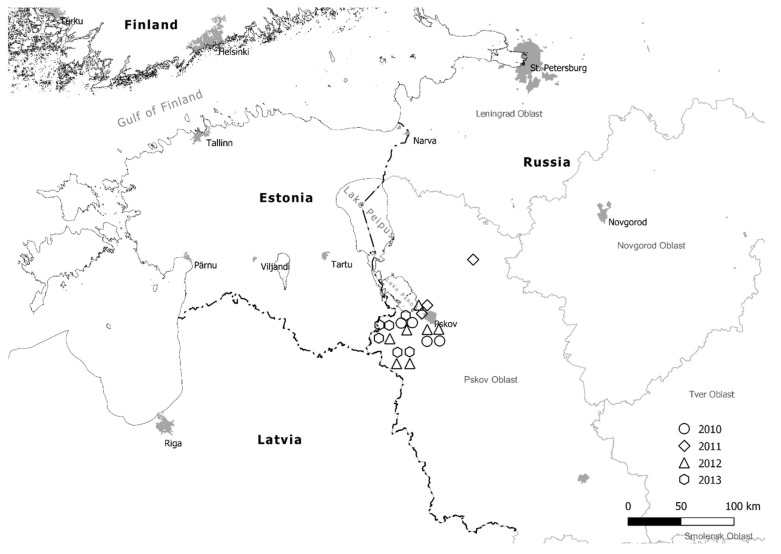
Map of Pskov region potato fields in north-west Russia where the isolates of *Phytophthora infestans* were collected during 2010–2013.

Potato leaves infected by *P. infestans* were collected over four years from 10 locations at the Pskov region in north-west Russia. A total of 238 isolates were collected from 20 sites located at Lakomtsevo (two sites), Pechory and Styagly in 2010; Strugi Krasnye, and Styagly (two sites) in 2011; Gusinets (two sites), Lezgi, Styagly (two sites), Tyamsha, and Zatrub’ye-Lebedy in 2012; and Bereznyuk, Bulatnovo, Gusinets (two sites), Lezgi, and Zatrub’ye-Lebedy in 2013 (Figure 1 and Table 1).

In 2010 and 2012, a majority of isolates were collected from small-scale (SSC) farm fields and a minority from large-scale conventional (LSC) production fields (Table 1). In 2011, all isolates were sampled from LSC fields, and in 2013, most isolates were collected from LSC fields and a minority from SSC fields (Table 1). Most of the samples were collected at the beginning of late blight season and some in mid-outbreak in each year (in July and August). At the end of the growing season (in August), samples were collected only in 2010 and 2011. In the early stages of the outbreak, approximately 10–15% of the leaf area of the infected plants and less than 10% of plants were infected with late blight. In the later stages, about 20–30% of the leaf area and more than 50% of the plants were infected. Metalaxyl-based fungicides were used for late blight control on LSC production fields. One to twenty-four isolates were cultured from each sampling site (Table 1). The plants selected for sampling were located at random distances from field edges. From each plant, leaves with single lesions were taken at random, isolations were carried out, and isolates were maintained using the methods described by Runno-Paurson et al. [29]. All phenotypic tests were carried out every study year immediately after the isolation was finished (October to January).

### 2.2. Phenotypic Assays

Mating types were determined by the method described in Runno-Paurson et al. [29]. The method involves growing each sampled isolate together with the appropriate tester strain (A1 and A2) in a Petri dish on rye B agar [30]. Plates were scored for oospore formation at the hyphal interface between the developing colonies after growth for 10–18 days at 16 °C in darkness. Isolates forming oospores on plates with the A1 mating type were classified as A2, whereas isolates that formed oospores with the A2 mating type were classified as A1. The tester isolates were 90,209 (A1) and 88,055 (A2), as described in Hermansen et al. [31].

Resistance to metalaxyl was tested using a modification of the floating leaflet method [31], as described by Kiiker et al. [27]. Leaf disks (14 mm diameter) were cut with a cork borer from leaves of five-week-old greenhouse-grown potato plants. The susceptible cultivar ‘Berber’ was used. Six leaf disks were floated abaxial side up in Petri plates (50 mm diameter) each containing 7 mL of distilled water or metalaxyl in concentrations of 10.0 or 100.0 mg L^−1^, prepared from technical grade mefenoxam (Syngenta experimental compound (metalaxyl-M), CGA 329351A). The inoculum was incubated for two hours at 4 °C to induce the zoospore release. The inoculation and trial incubation was done as described by Runno-Paurson et al. [29]. The isolates were rated resistant if they sporulated on leaf disks in 100 mg L^−1^ metalaxyl [31]. Those sporulating on leaf disks in a metalaxyl concentration of 10 mg L^−1^, but not on leaves floating in 100 mg L^−1^ were rated intermediate, and those sporulating only in water were rated sensitive.

### 2.3. DNA Extraction and Microsatellite (SSR) Marker Analysis

Pure-culture *P. infestans* isolates were grown on rye B agar plates for 2–4 weeks at 17 °C in the dark. Mycelium was scraped off from Petri plates with a sterile scalpel blade and frozen at −80 °C. DNeasy Plant Mini Kit (QIAGEN, Manchester, UK) was used to extract DNA following the manufacturer’s instructions. Genotyping of 141 isolates was performed at The James Hutton Institute (Dundee, Scotland, UK), following Li et al.’s [28] SSR marker 12-plex method with some modifications by Kiiker et al. [27,32]. One primer for each locus was labeled with different fluorescent dyes (6FAM; NED; VIC; PET, Applied Biosystems, Waltham, MA, USA). PCR reactions were performed in a volume of 12.5 µL consisting of 1× Type-it Multiplex PCR Master Mix (QIAGEN Type-it Microsatellite PCR Kit, Cat. No. 206243), primers for each locus at optimal concentrations ranging from 0.32 to 0.035 µM and 1 µL of template DNA (approximately 20 ng µL^−1^ and within a range of 5–200 ng µL^−1^). Amplification reactions were carried out on a Bio-Rad T100 Thermal Cycler (Watford, UK) under the following conditions: 95 °C for 5 min followed by 28 cycles of 95 °C for 30 s, 58 °C for 90 s, and 72 °C for 20 s, and a final extension at 60 °C for 30 min. PCR products were diluted 0–100 times depending on the initial DNA concentration in the PCR reaction mix. The diluted PCR product (0.6 µL) was added to a mix containing 10.14 µL of deionized formamide (Hi-Di Formamide, Applied Biosystems) and 0.06 µL of GeneScan-500LIZ standard (Applied Biosystems). A detailed protocol is provided on the EuroBlight website (https://agro.au.dk/forskning/internationale-platforme/euroblight/protocols) (accessed on 10 April 2022). The fluorescent-labeled PCR products were analyzed using an ABI3730 DNA Analyzer (Applied Biosystems) according to the manufacturer’s instructions. The size of the alleles was determined using GeneMapper v3.7 (Applied Biosystems) software.

### 2.4. Data Analysis

*Phytophthora infestans* isolates collected from 20 sites in Pskov region of Russia during 2010–2013 were analyzed for mating type (238 isolates), metalaxyl response (189 isolates), and a representative selection of 141 isolates was chosen from of 238 *P. infestans* isolates for SSR marker genotyping, keeping the A1 and A2 mating type frequencies within fields and in the whole population about the same as previously published by Runno-Paurson et al., 2016 [26]. Differences in the prevalence of the two mating types of *P. infestans* isolates between years, study sites, locations, and agricultural management practices were tested using a logistic analysis (GENMOD procedure in SAS [33]) with a multinomial response variable (A1, A2, or both), when ordinary chi-square assumption was not met. Here we ran a Monte Carlo simulation to overcome any problems with assumptions in chi-square to give accurate estimates of significance. The procedures were used to examine the differences in the resistance to metalaxyl (a multinomial response variable: resistant, intermediate, and sensitive) between years, sites, locations, agricultural management practices, and also between different mating types.

Nei’s [34] measure of allelic diversity and the number of alleles were estimated using the package *poppr* (ver. 2.9.3) [35] in R (ver. 3.6.2) [36]. GenAlEx [37] was used to calculate allele frequencies. The number of multi-locus genotypes (MLGs), and expected number of MLGs after rarefaction (eMLG), richness, and evenness (E5) were estimated using the package *poppr*. Simpson’s index of genotypic diversity (λc; the probability that two randomly selected genotypes are different), corrected for sample size (N) by multiplying lambda by N/(N−1), was also calculated using the package *poppr* for the locations, years, and agricultural management practices. Simpson’s index (λ) was also used to measure the gene diversity of the SSR loci. To eliminate the bias imposed by the large asexual reproductive capacity (clonal genotypes), a clone corrected dataset [38,39] was constructed using *poppr* package to include only one representative isolate of each multilocus genotype [40,41,42]. The data was clone-corrected before the analysis of Hardy–Weinberg equilibrium (HWE), linkage disequilibrium (LD), and test of AMOVA (with the ade4 implementation of AMOVA) also using *poppr*. As many genetic distance measures are not always euclidean, this was corrected for before analysis of AMOVA. *Poppr* automates this with three methods implemented in ade4 [43]. An analysis of molecular variance (AMOVA) tests whether *P. infestans* populations were genetically differentiated and assesses how genetic variation was distributed among and within populations based on 1000 permutations. A linkage disequilibrium (LD) analysis was used to infer the reproductive strategy of *P. infestans* based on the index of association (*I_A_*) and standardized index of association (r¯d). The null model for *I_A_* is random recombination [44]. They were estimated based on 999 permutations [45] to simulate random mating and implemented using *poppr* for clone-corrected datasets. As the index of association is dependent on the sample size of loci, we removed this dependency and used the standardized index of association (r¯d) that accounts for the number of loci sampled and is less biased [46]. The population structure of *P. infestans* was investigated using discriminant analysis of principal components (DAPC). DAPC can determine any clusters of genetically related isolates, and display differences between clusters of genetically distinct individuals while minimizing variations within clusters [47]. The optimal number of clusters was assessed by Bayesian information criterion (BIC) using the ‘find. cluster’ function. DAPC relies on data transformation, using PCA as a prior step to the discriminant analysis (DA). Without implying a necessary loss of genetic information, this transformation allows DA to be applied to any genetic data [48]. The number of principal components to retain in the analyses, was determined by a stratified cross-validation, as implemented in adegenet [47]. Furthermore, a DAPC-based structure analysis was constructed to examine the probabilistic assignment of individuals to each group based on the hypothesized ten locations, four years, and two agricultural management practices, respectively, which was visualized by DAPC analysis. An α-score optimization was used to determine the optimal number of principal components to retain [47]. All these procedures were conducted and visualized in the R package *adegenet* [47]. The DAPC approach has been reported to produce consistent results [48,49], or even perform generally better and computationally less expensive (faster) than STRUCTURE [48] in the analysis of the genetic structure of populations [48,49] and identifying prominent differences [49]. Interestingly, DAPC also allows for a probabilistic assignment of individuals to each group, as in STRUCTURE software [48]. In addition, the DAPC is not based on any assumption about the underlying population genetics model such as Hardy–Weinberg equilibrium or linkage equilibrium, common to other methods used to detect population structure, which are often difficult to verify and can restrict their applicability [48]. In another method of analysis of genetic structure, Bayesian clustering, which involves K-means clustering in combination with bootstrapped dendrograms, was used to create a neighbor-net phylogenetic tree using a boot with Bruvo’s genetic distance to investigate the population genetic structure [50]. Finally, genetic relatedness among multilocus genotypes (MLGs) was assessed by calculating Bruvo’s genetic distance using *poppr* (function bruvo. msn) and visualized using a minimum spanning network (MSN) [35].

The coefficient of genetic differentiation (GST) at population level was estimated as GST = (*H*_T_ − *H*_S_)/H_T_, where *H*_T_ corresponds to the total gene diversity, and *H*_S_ corresponds to the gene diversity within sub-populations [51,52]. Gene flow is defined as the average per generation of migrants transferred across populations and it is estimated indirectly based on the formula: Nm = 0.5 (1 − GST)/GST [53]. If Nm < 1, then the population continues to differentiate, and if Nm ≥ 1, there will be little differentiation among populations, as genetic differentiation between populations will be hindered by gene flow [54]. There is another genetic differentiation measure called Jost’s D [55]. *G*_ST_ and *D* measure differentiation in very different ways. Jost [55] believed that *G*_ST_ and its relatives do not really measure differentiation and that *G*_ST_ depends on average within-population heterozygosity (*H*_S_), which prevents *G*_ST_ from taking values larger than average homozygosity. Though *G*_ST_ is mathematically constrained by *H*_S_, there is no such constraint on *D*, implying that *D* can take any value in the range 0–1 regardless of *H*_S_ [56]. However, *D* still shares the same problems of dependence on *H*_S_ and mutation rate as *G*_ST_ and its relatives, and the problems are even more pronounced for *D* than for *G*_ST_. The author concluded that *G*_ST_ is still a very useful measure of genetic differentiation [56]. Therefore, we used the *G*_ST_ as a measure of genetic differentiation.

## 3. Results

### 3.1. Mating Type

**Figure 2:**
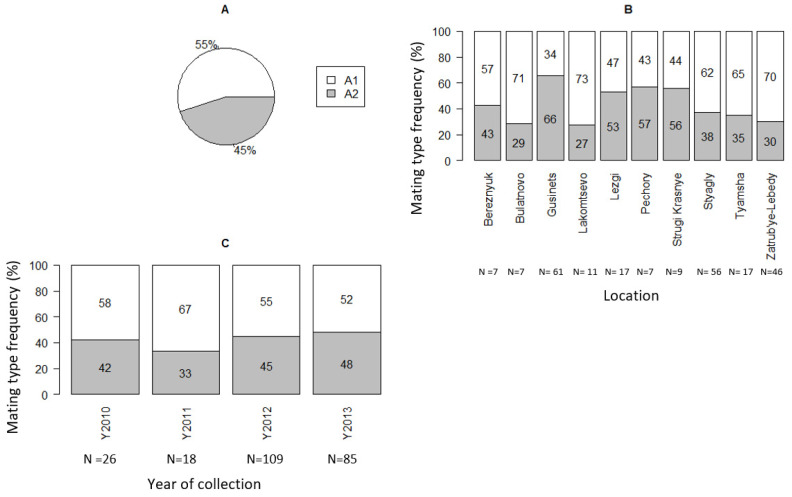
Mating types (A1, A2) frequency among P. infestans isolates collected from Pskov region in north-west Russia during 2010–2013. (**A**) For all isolates. (**B**) For locations. (**C**) For sampling years.

The overall distribution of A1 and A2 mating type isolates were nearly equal to 1:1 as, among the 238 isolates, 131 (55%) were the A1 mating type and 107 (45%) were the A2 mating type (Table 1 and Figure 2A). Both A1 and A2 mating types were found in all four study years. There were significant differences in the frequency of A1 and A2 mating types between sampling sites (χ^2^ = 38.62, df = 38, *p* < 0.01) as well as between locations (χ^2^ = 19.73, df = 18, *p* < 0.02), where a higher frequency of the A2 mating type was found among isolates collected from Gusinets, Pechory, Strugi Krasnye, and Lezgi (Figure 2B). The frequency of A1 and A2 mating types varied with the year (Figure 2C), however, there were no statistically significant differences between study years (χ^2^ = 1.426, df = 3, *p* = 0.699). In 2011, the frequency of A2 was low (33%), possibly due to the low number of isolates tested, compared to other years. Both mating types were present at 17 of the 19 sites (90%) where more than one isolate was tested. There were no significant differences between different agricultural management practices (large-scale conventional (LSC) fields vs. small-scale conventional (SSC) fields; χ^2^ = 2.47, df = 1, *p* = 0.16).

### 3.2. Metalaxyl Resistance

**Figure 3:**
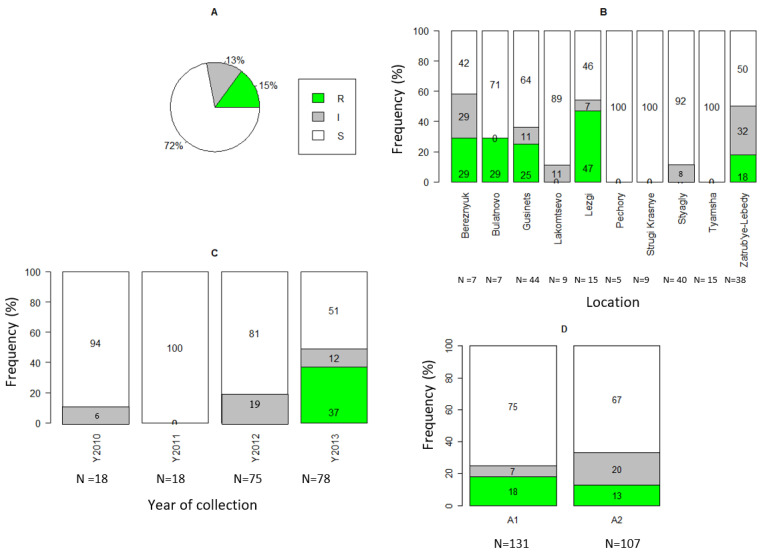
Metalaxyl resistance among *P. infestans* isolates collected from Pskov region in north-west Russia during 2010–2013. (**A**) For all tested isolates. (**B**) For locations. (**C**) For sampling years. (**D**) For A1 and A2 mating type. R: resistant, I: intermediate, S: sensitive. Note: Number in parenthesis in Figure 3B is the number of isolates.

Of the 189 isolates tested, 29 (15%) were classified as resistant, 24 (13%) intermediate, and 136 (72%) as sensitive (Figure 3A). Highly significant differences were found between sampling sites (χ^2^ = 90.42, df = 38, *p* < 0.0001) and locations (χ^2^ = 55.05, df = 18, *p* < 0.0001). Among the isolates, metalaxyl-sensitive phenotypes predominated in Pechory, Strugi Krasnye, and Tyamsha with 100%, and in Stagly with 92% (Figure 3B). The metalaxyl-sensitive isolates dominated in most of the studied years (Figure 3C). The proportion of metalaxyl-sensitive strains varied from 100% in 2011 to 51% in 2013. Metalaxyl-resistant strains only appeared in 2013 with a frequency of 37% (Figure 3C). The association between the metalaxyl response and years was highly significant (χ^2^ = 55.25, df = 6, *p* < 0.0001). Significant differences were found between different agricultural management practices (χ^2^ = 18.24, df = 2, *p* < 0.001), where a higher proportion of resistant isolates (25%) were found on LSC fields compared to SSC fields (9%). Metalaxyl-sensitive isolates dominated on SSC fields (83%), while the frequency of sensitive isolates on LSC fields was considerably lower (54%). All three categories of responses to metalaxyl were found in both A1 and A2 mating types (Figure 3D). The majority of tested A1 and A2 isolates were sensitive (75 and 67%, respectively) (χ^2^ = 6.93, df = 2, *p* = 0.03).

### 3.3. SSR Polymorphisms

Among the 141 isolates characterized with SSR markers, 50 alleles were detected at 12 loci, which were all polymorphic. They displayed allelic diversity among the isolates, with 2 (SSR6, Pi70, and SSR2) to 8 alleles (G11 and SSR4) per locus, and an average of 4.17 ± 0.65 alleles (Table 2). The minimum allele size was 132 bp, while the maximum size was 355 bp. Likewise, the allele frequency ranged from 0.004 to 0.950 (Table S1). Gene diversity varied from 0.094 (locus Pi70) to 0.799 (G11), with an average of 0.532 ± 0.06. Six out of twelve loci (G11, Pi04, Pi4B, Pi63, SSR4, and SSR11) were more diverse with a value above the overall mean (Table 2). *He* estimates the mean expected heterozygosity at the locus. The locus Pi70 only had two alleles, and the locus also had a low genetic diversity (0.094), and low expected heterozygosity (0.095). This is a sign that this might be a phylogenetically uninformative locus, while the loci G11 (*He* = 0.802 and genetic diversity = 0.799) and SSR4 (*He* = 0.769 and genetic diversity = 0.766) were the most informative loci with 8 alleles per locus (Table 2). Of loci with more than two alleles, Pi04 was found to be the most evenly distributed (evenness), followed by Pi4B (Table 2). Not all loci were in the Hardy–Weinberg equilibrium with respect to each location, year, or agricultural management practice (Figure S1), as expected in naturally evolving non-equilibrium populations. Overall, of the isolates, for each locus, four loci (D13, G11, PiO4, and Pi70) were not in the Hardy–Weinberg equilibrium (Pr. exact < 0.05) (Table 2). Eighty-three multilocus genotypes (MLGs) were identified.

### 3.4. Population Structure

**Figure 4:**
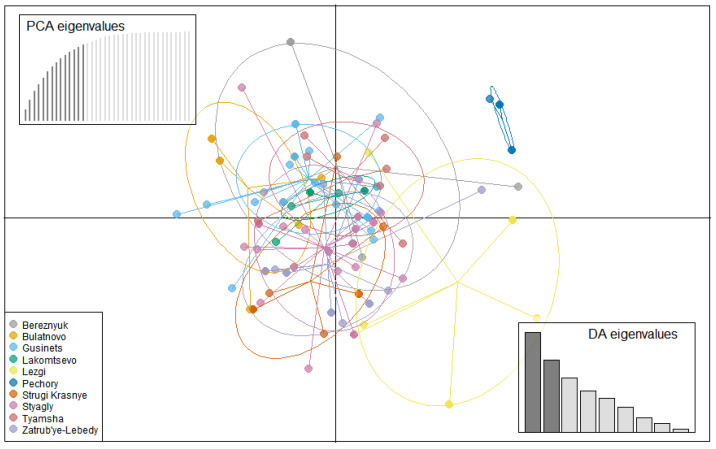
A discriminant analysis of the principal components (PC) scatterplot showing clustering of *P. infestans* isolates according to locations (non-clone corrected). Clusters representing each location are indicated by the color-coded ellipses. Scatterplots represent the distribution of individuals along the first two linear discriminants. Dots represent isolates’ multilocus genotypes (in each population). The cross-validated number of the PCs used for the discriminant analysis is shown in the bar plots on the top left of the scatterplot. Indicated at the bottom right corner is the discriminant analysis eigenvalues, respectively. A total of 14 PCs explained 99.4% of the variation.

**Figure 5:**
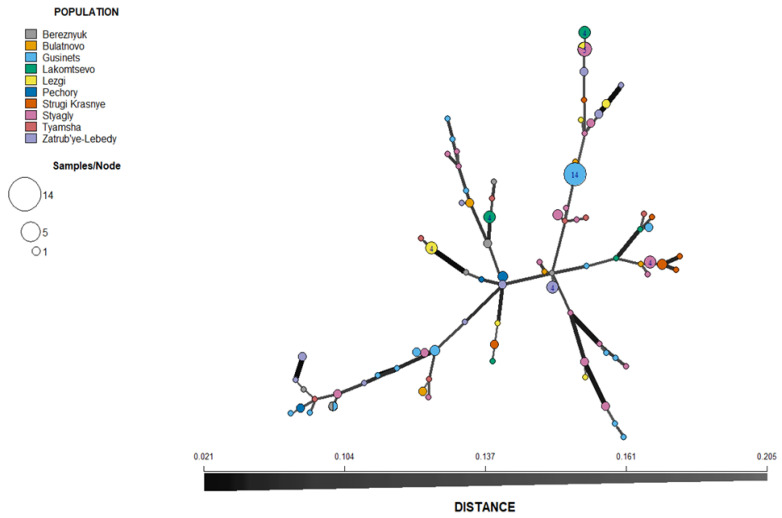
Minimum spanning network (MSN) inferred from simple sequence repeat (SSR) markers of *P. infestans* collected from ten locations (non-clone corrected) of Pskov region in north-west Russia during years 2010–2013. Each node represents multilocus genotypes (MLGs) and the size is proportional to the number of individuals sharing the same SSR allele profile. The thickness of the lines represents the Bruvo’s genetic distance between two nodes (the further the genetic distance the lighter the color and thinner the line). MLGs from the same location are color-coded.

**Figure 6:**
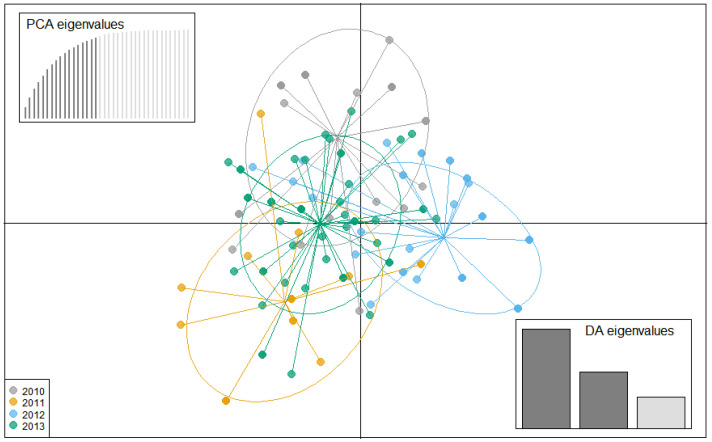
A discriminant analysis of the principal components (PC) scatterplot showing clustering of *P. infestans* isolates according to years (non-clone corrected). Scatterplots represent the distribution of individuals along the first two linear discriminants. Clusters representing each year are indicated by the color-coded ellipses. Dots represent isolates (in each population). The cross-validated number of the PCs used for the discriminant analysis is shown in the bar plots on the top left of the scatterplot. Indicated at the bottom right corner is the discriminant analysis eigenvalues, respectively. A total of 17 PCs explained 99.4% of the variation.

**Figure 7:**
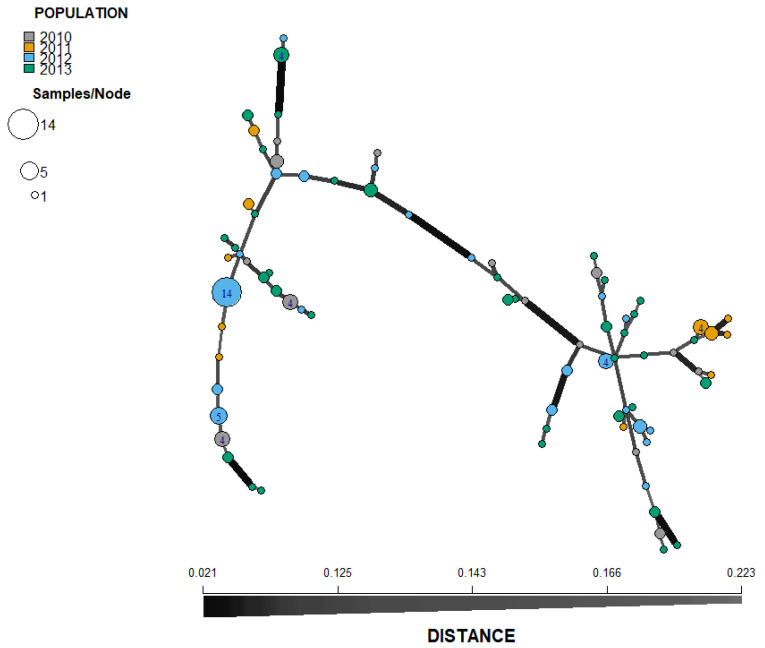
Minimum spanning network (MSN) inferred from simple sequence repeat (SSR) markers of *P. infestans* (non-clone corrected) collected from Pskov region in north-west Russia during four year period (2010–2013). Each node represents MLGs and the size is proportional to the number of individuals sharing the same SSR allele profile. The thickness of the lines represents the Bruvo’s genetic distance between two nodes (the further the genetic distance the lighter the color and thinner the line). MLGs from the same year are color-coded.

**Figure 8:**
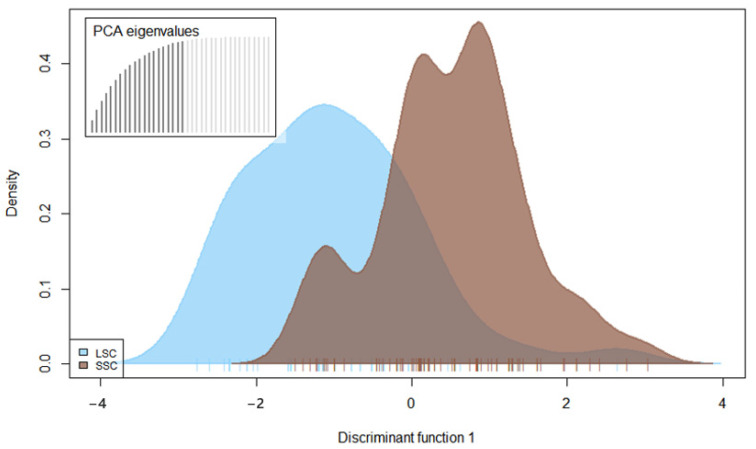
A discriminant analysis of the principal components (DAPC) of *P. infestans* isolates (non-clone corrected) collected on large-scale conventional farm (LSC) and small-scale farm (SSC). DAPC fails to discriminate between populations. Colors indicate population. The cross-validated number of the PCs used for the discriminant analysis is shown in the bar plots on the top left of the density plot. A total of 20 PCs explained 99.1% of the variation.

**Figure 9:**
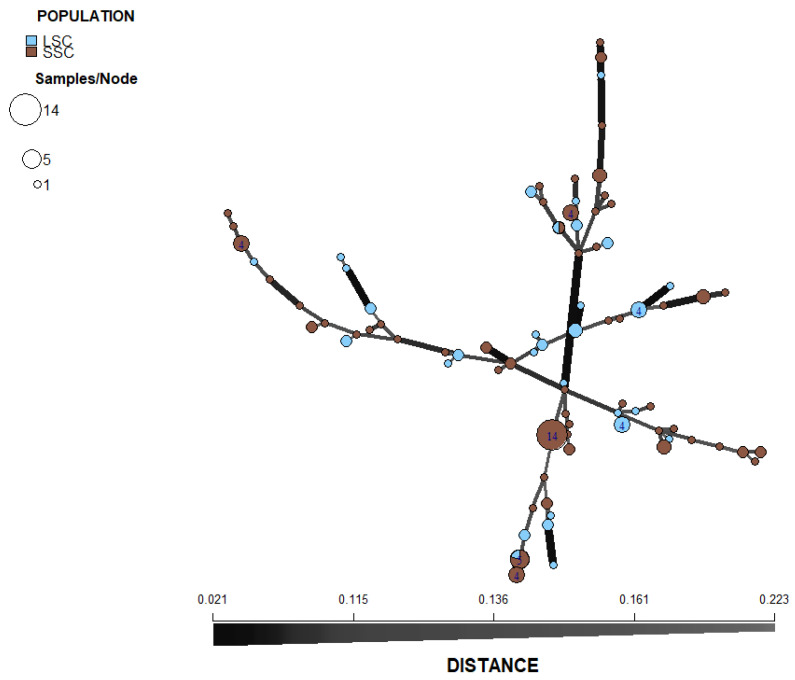
Minimum spanning network (MSN) inferred from simple sequence repeat (SSR) markers of *P. infestans* (non-clone corrected) collected on large-scale conventional farm (LSC) and small-scale farm (SSC). Each node represents multilocus genotypes (MLGs) and the size is proportional to the number of individuals sharing the same SSR allele profile. The thickness of the lines represents the Bruvo’s genetic distance between two nodes (the further the genetic distance the lighter the color and thinner the line). MLGs from the same agricultural management practice are color-coded.

With the exception of isolates from Pechory, discriminant analysis of principal components (DAPC) performed with the SSR datasets showed no clear pattern of grouping among isolates of *P. infestans* according to location (Figure 4). Membership probability for each isolate, as determined by DAPC, was used to visualize the distribution of cluster assignments within and between populations. Membership probability assignments did not reveal high levels of population differentiation because each genotype had similar probabilities of membership with each cluster and the admixture of isolates with shared alleles from different locations was evident in each location (Figure S2). However, most isolates from Pechory had a higher probability of assignment to a cluster separate from any other location, similar to the observation in Figure 4. Similarly, the minimum spanning network (MSN), based on Bruvo’s genetic distance (Figure 5), did not cluster isolates according to their location (MLGs from the different locations were interspersed throughout the network, rather than forming a distinct single cluster) and was consistent with the DAPC analysis. Gusinets was mostly dominated by a single MLG (Figure 5). This MLG had 14 isolates where 4 and 10 isolates were of the A1 and A2 mating types, respectively. All of the 14 isolates were from SSC, nine metalaxyl-sensitive, one metalaxyl-intermediate, and the others not determined, and all from the year 2012. There were only two examples of a single MLG common to two locations. In one case, an MLG with a single isolate from Lezgi (A1 mating type, from LSC and metalaxyl-sensitive) matched four isolates from Styagly (all A2 mating type, and from SSC, three metalaxyl-intermediate and one metalaxyl-sensitive), all sampled in 2012. Another MLG was common to two isolates, one from Bereznyuk (A1 mating type, from LSC and metalaxyl-resistant) and one from Gusinets (A2 mating type, from SSC and metalaxyl-sensitive), both sampled in 2013. All other MLGs with more than one representative isolate were sampled from the same location in the same year. From the 83 MLGs (with two MLGs that shared isolates from different locations), 54 (65%) were unique as they appeared only once, with Gusinets and Styagly having the highest number (12 each), while Pechory had only one unique MLG. Figure S3 shows the alleles that played a role in partially separating Pechory from other locations. These were alleles 142 and 156 in locus D13, alleles 264 and 266 in locus Pi02, and alleles 296 in locus SSR4. While alleles 142 and 156 in locus D13, and alleles 264 and 266 in locus Pi02 were absent in Pechory, only allele 296 of locus SSR4 was shared between Pechory, Gusinets, Lakomtsevo, Lezgi, Styagly, Tyamsha, and Zatrub’ye-Lebedy. No association between the genotype and year of sampling was observed in the DAPC (Figure 6), MSN (Figure 7), or membership probability (Figure S4) analyses. No MLG was sampled in more than one season. A particular MLG with 14 isolates dominated in 2012, as also shown in the MSN (Figure 7). No association between the genotype and management practices was observed in the DAPC (Figure 8), MSN (Figure 9), or membership probability (Figure S5) analyses. The only cases with MLGs in common between management practices were the two already mentioned under the location, where a single isolate from Lezgi (A1 mating type, from LSC and metalaxyl-sensitive) matched four isolates from Styagly (all A2 mating type, and from SSC (three metalaxyl-intermediate and one metalaxyl-sensitive)), all sampled in 2012. A look at the distribution of the MLGs for each agricultural management practice showed there are more unique MLGs in the SSC in comparison to the LSC population. About 39 MLGs out of 55 MLGs (71%) were unique in SSC, while in LSC, 18 MLGs out of 30 MLGs were unique (60%). In the clone-corrected data, there was also no clear population structure (samples did not cluster exclusively by population) as revealed in the DAPC, MSN, population membership structure analysis, and a neighbor-net phylogenetic tree for locations, years, and agricultural management practices (Figures S6–S9).

### 3.5. Population Genetic Differentiation and Linkage Analysis

The AMOVA revealed that no significant population differentiation was found when populations were grouped by location, years, and agricultural management practices (*p* = 0.207, *p* = 0.226, and *p* = 0.238, respectively; Table 3), which supports the DAPC and MSN result. Most variation was found within the location, years, and agricultural management practices, accounting for 94.7, 97.4, and 98.9% of the genetic variations in the clone-corrected data, respectively. Variations attributable to the difference among the populations accounted for only 5.3, 2.6, and 1.1% of the total genetic variation, indicating very low levels of differentiation at the scale of the population for location, years, and agricultural management practices, respectively. The null hypothesis of no linkage among loci was rejected at *p* > 0.05 and indicates sexual reproduction can occur in the population. The test of a standardized index of association (r¯d) supported random mating for locations, years, and agricultural management practices, respectively, for the clone-corrected data set (Table S2), indicating sexual reproduction could be expected. Using Nei’s gene diversity statistics, the genetic diversity of the locations was explored further. The analysis of the occurrence of genetic variation indicated that the mean gene diversity within populations (*H*_S_) and total gene diversity (*H*_T_) were 0.478 and 0.533, respectively (Table 4). The genetic diversity coefficient among the populations (GST) was 0.102. The GST value less than 1 suggests a high level of genetic similarity and low genetic differentiation among the populations. The gene flow (Nm) among the populations was 4.402. The high level of gene flow among the population is consistent with the low genetic differentiation among the populations.

### 3.6. Multilocus Genotype and Genotypic Diversity

The number of expected multilocus genotypes, corrected for sample size (eMLG), was used to compare the genotypic richness among the locations, years, and agricultural management practices. Zatrub’ye-Lebedy was genotypically richer (9.36) than the other locations (Table 4), while the genotypic richness of Lezgi was the least with 3.00. Likewise, the year 2013 was genotypically richer (15.9) compared to 2012 (11.1), 2011 (11.0), and 2010 (12.0), while for agricultural management practices, SSC was genotypically richer (33.0) compared to the LSC (30.0) for eMLG, and 30 (LSC) and 55 (SSC) for MLG, respectively. The Simpson’s index, corrected for sample size (λc), indicated that genotypic diversity was very high in all the locations, varying between 0.73 in Pechory to 1.00 in Tyamsha, the most genotypically diverse location (Table 4). Throughout the years, λc was also very high, ranging from 0.90 in the year 2012 to 0.99 in the year 2013, the most genotypically diverse year. λc for 2010 was 0.94, while for 2011, it was 0.93. For agricultural management practices, there were a high genotypic diversity in large-scale farms (*λc* = 0.98) and small-scale farms (*λc* = 0.97). The result of genotypic evenness (*E_5_*) indicates that the MLGs observed in Tyamsha were equal in abundance with an *E_5_* value of 1.0 (Table 4), while other locations were also closer to an equal abundance of genotypes, but Gusinets had the lowest genotypic evenness (*E_5_* = 0.48). MLGs observed in the year 2013 were closest to equal the genotype abundance (*E_5_* = 0.89) compared to the year 2010 (*E_5_* = 0.83) and the year 2011 (*E_5_* = 0.84), while the year 2012 was more dominated by a single genotype (*E_5_* = 0.58). A comparison of agricultural management practices showed that MLGs were more evenly distributed (*E_5_* = 0.87) in LSC compared to SSC (*E_5_* = 0.61). Overall, the value of genetic diversity across the location was moderately high, *He* = 0.533 (Table 4), except for in Pechory (*He* = 0.234). The gene diversity across the years was also high, ranging from 0.489 in the year 2012, 0.507 in 2010, and 0.521 in 2011 to 0.551 in the year 2013, with an overall value of 0.533, and a mean of 0.517 ± 0.01. The populations of *P. infestans* isolates had high levels of genetic diversity in both LSC (*He* = 0.532) and SSC (*He* = 0.531), with an overall value of 0.533, and a mean of 0.532 ± 0.00.

## 4. Discussion

In-depth knowledge of the traits, composition, and dynamics of a changing *P. infestans* population will help to improve late blight control, as it could provide information on the improved choice of control strategy and/or fungicides, as well as direct resistance breeding efforts [11]. Russia is one of the most important potato producers and consumers in Europe. However, unlike the common potato growing practices in most European countries, the majority of the tuber harvest is produced by small households with small average plot sizes and without the application of late blight control measures. Thus, in the present study, the genotypic and phenotypic diversity of the *P. infestans* population were investigated in the unstudied Pskov region of Russia, to determine how the pathogen populations differed by location, year, and agricultural management practices.

### 4.1. Population Differentiation and Gene Flow

Gene flow is an important evolutionary force that positively influences the adaptive responses of plant pathogens to changes in the environment and plant disease management strategies [57]. Pathogen population divergence may occur as a result of a genetic drift and local adaptation to increase the relative fitness in local environments [58]. A migration rate greater than one is sufficient to prevent significant divergence between populations or prevent local adaptation, and migration is more important than genetic drift [53]. The processes of extinction and recolonization (balance between genetic drift and migration), anthropogenic activities, such as the movement and exchange of infected plant material, as well as alternate hosts outside of the growing season either alone or in combination are some of the mechanisms that can enhance gene flow [40,53,58,59]. An insight into the structure of the *P. infestans* populations from different locations is valuable in enhancing the understanding of the biology of the pathogen and the potentially adaptive genotypic diversity in the species. The structural analysis indicated an admixture of isolates among the populations. No separation of isolates into distinct isolated subpopulations by location was observed. This may be attributed to gene flow among the locations, which is reflected by the high migration rate estimates. A high genetic exchange or high gene flow will lead to low genetic differentiation, as evidenced by the GST value. The migration may be due to the long-distance dispersal of spore and/or anthropogenic activities, such as the movement and exchange of infected plant material [60]. The late blight pathogen is cosmopolitan in distribution, and the spores are known to be disseminated over long distances by wind and via the movement of infected materials [60]. The genetic diversity of *P. infestans* and the evolutionary forces responsible for its diversity has also been investigated in China using molecular population genetics approaches [61]. The authors noticed the considerable level of gene flow between populations, as well as the direction of gene flow, primarily from north to south, which corresponds to the route of potato seed transportation, suggesting a role of human activities in the dispersal of *P. infestans*. This illustrates an example of a source–sink dynamic of the populations and diversification patterns shaped by gene flow. However, on a global scale, the geographic barrier and minimum cross-border trade can disturb the gene flow between pathogen populations, even between neighboring countries. There are reported cases of a lack of gene flow of *P. infestans* populations between China and India, which may be attributed to the presence of the Himalaya Plateau and the minimum cross-border trade of potato and tomato products between these neighboring countries [57].

### 4.2. Evidence of Sexual Reproduction

We detected both the A1 and A2 mating types in all locations and all years and the overall ratio of *P. infestans* mating types was close to 1:1, with no significant differences in the proportion between years. This observation is common for a sexually reproducing population and consistent with the local production of oospores [26,62]. However, a sexually reproducing population may not be judged alone by the presence of both A1 and A2 mating strains and the ratio because *P. infestans* can reproduce clonally even if both mating types are present in a region or location. Therefore, other pieces of evidence were sought to confirm this observation [63]. We failed to reject the null hypothesis of no linkage among markers, which is expected of a freely recombining sexual population. In addition, the lack of population structure, as revealed by DAPC, the structural analysis of population membership, a minimum spanning network, AMOVA, and a neighbor-net phylogenetic tree are indications that novel genotypes in *P. infestans* are produced by sexual recombination, giving high levels of genetic diversity [64]. A very high proportion of unique multilocus genotypes identified is also consistent with a more important role of the sexual cycle and the formation of oospores. This results in high levels of recombination and possibly with a limited spread of these genotypes through the asexual cycle [65]. The result is not surprising as stress factors such as cold winters, as observed in Russia, the Baltics, and Scandinavia, favor the production of oospores which results in a population more influenced by sexual reproduction as oospores are more durable and can survive in the soil [27,32,66]. Until this work, evidence of sexual reproduction among *P. infestans* in some regions of Russia had not been documented [13,20,67]. Sexual reproduction also characterizes the *P. infestans* population of Scandinavia, the Baltic states, eastern Europe (including Russia), and Mexico [13,23,26,32,62,68,69,70].

### 4.3. Genotypic Diversity of P. infestans

The genotypic diversity among the Russian *P. infestans* populations was very high. This was also combined with many observed unique multilocus genotypes (MLGs). The high genotypic diversity with SSR markers and the RG57 fingerprints also confirms the previous result from Moscow [13,71] and some regions of Russia (Astrakhan, Kostroma, Nizhnii Novgorod), where nearly all isolates were unique [13,20]. The high variation could also be influenced by the shorter geographic distances among the populations, allowing random mating and outbreeding. This is also shown by a low genetic differentiation among these populations. High genotypic diversity through sexual reproduction and oospores’ formation is common in neighboring countries such as Finland and the Baltic region [26,32,68], but differs from essentially clonal populations in western regions of Europe [72,73,74], and in the United States and South America [75,76], Asia [61,77], and Africa [78,79]. However, recent rapid local and global genotype shifts have occurred due to pathogen migration with tuber trade [80,81]. Most of the markers were informative, except SSR Pi70 which showed almost no diversity, as observed in previous studies [27,62]. High levels of genetic diversity and linkage equilibrium among loci may contribute to higher levels of fitness in pathogens. This, in part, can also contribute to the evolution of novel traits which can hinder the effectiveness of control measures, such as overcoming novel blight resistance genes or conferring insensitivity to fungicide active ingredients [82]. Dominant MLGs were present in subpopulations (Gusinets and Styagly), suggesting this is related to a local sampling site. Therefore, these genotypes could also be included in screening and selection protocols to identify tolerant or resistant plant material. Some genotypes were shared among locations, highlighting the occurrence of gene flow among populations within the Pskov region, or showing that there may be a common source of pathogen inoculum [83]. Interestingly, the gene flow among populations is estimated, which suggests that the migration and gene flow among the populations promotes an admixture among isolates from the different locations. This can influence the genotypic diversity among the population. The absence of shared genotypes between years suggests a population constantly evolving over time. It is, therefore, necessary to constantly monitor the population changes of *P. infestans*, which will help to identify new genotypes. Crop rotation rules are not usually strictly adhered to by small-scale farmers, as they tend to grow potatoes every year on the same field with inadequate control of late blight. Thus the risk of oospore-derived infections is high, resulting in a genotypically diverse inoculum in fields, which is confirmed by the current results of several unique MLGs observed in small conventional fields.

It is interesting to note that some of the genotypes with isolates from the same location, as well as genotypes with isolates from different locations that had similar SSR profiles were of different mating types. We have proposed a mating-type switching at the mating-type locus as an explanation for this observation. Mating-type switching had been observed in *Phytophthora* spp. [84] and other organisms, such as yeast [85]. This assumed reversible change may be induced by stress from either fungicidal treatment or other sources. For instance, it was highlighted by others that ploidy levels in *P. infestans* strains can vary and the triploids can change to a diploid genome constitution upon stress [86]. It is unlikely that the genome contains both mating-type loci such that either or both can be switched on depending on the prevailing environmental condition. On the other hand, the 12 SSR markers may not be sufficient to genetically discriminate the genotypes, hence other methods, such as restriction site-associated DNA sequencing (RAD-seq) that generates large marker sets, are advised [63].

### 4.4. Metalaxyl Sensitivity and Agricultural Management Practices

For effective disease management in some European countries, repetitive chemical spraying, more than ten times per season, needs to be applied per season [87]. Furthermore, several active ingredients have to be used to reduce the emerging likelihood of insensitivity to commonly used active ingredients [87,88]. Multiple fungicide applications on an infected crop can increase the selection pressure on the local pathogen population, and if an insensitive clonal variant emerges, it will dominate and reduce the genotypic richness and diversity [89,90,91], as well as its genetic diversity [50,92]. Contrarily, less frequent or no fungicide application rates, as may occur in small-scale farms, reduce the likelihood of such a selection pressure, enabling metalaxyl-sensitive and less aggressive genotypes to survive and flourish in these settings [91]. Furthermore, in small-scale farms, many ‘heritage’ varieties, developed before blight resistance was a common breeding objective, are cultivated, in addition to commercial varieties, creating a wider range of varieties than in commercial systems. With less frequent or no fungicide application, this again provides an environment in which less virulent *P. infestans* genotypes can flourish in addition to those that infect commercial crops [91]. Altogether, these maintain the genotypic richness that comes from the oospore population in the soil [25,93]. This may explain the higher genotypic richness observed in small conventional fields in the Pskov region, with more MLGs. Furthermore, the predominance of metalaxyl-sensitive isolates observed in small conventional fields compared to large-scale conventional farming reflects the significant effect of a cultivation system on the selection pressure of a pathogen population, altering the sensitivity levels to active ingredients [74,89,90,91]. Runno-Paurson et al. [94], recorded a higher proportion of metalaxyl-resistant isolates in large conventional farms compared to small conventional farms, which was also observed in this study. A similar result was also reported by Brylinska et al. [92] and Cooke et al. [95]. In Russia, fungicides are only applied in big potato growing enterprises and not used in small-scale farms [18]. In Nordic and Baltic countries, the proportion of metalaxyl-resistant isolates has decreased in recent times, due to the limited use of fungicide, compared with the early 1990s [27,32,93,96].

It is clear from this study that isolates sensitive to metalaxyl prevailed in the population, as also observed in Moscow and other Russian populations of *P. infestans* [21]. These findings corroborate results from other studies from Estonia [27], Latvia [25], Poland [62,97], Lithuania [93], the Czech Republic [98], and Nordic countries [96]. However, the predominance of metalaxyl-resistant strains has been reported in several European populations, such as the Republic of Ireland, Northern Ireland, France, and Cyprus [74,99,100,101], which is mainly due to the spread of the mefenoxam-insensitive genotype EU_13_A2. The higher proportion of metalaxyl-sensitive strains of *P. infestans* observed may have resulted from moderate use of metalaxyl fungicide on the fields from which the samples were collected [25,27].

## 5. Conclusions

Sexual reproduction likely takes place in the *P. infestans* population in the Pskov region. This is supported by the presence of both mating types in all locations studied and in several fields where multiple samples were taken, as well as evidence from the genetic analysis of the population structure, tests of linkage among loci, and other analyses of the genotypic data. Resistance to metalaxyl was present, although the metalaxyl-sensitive isolates predominated. The genotypic and phenotypic variation is consistent with regular sexual reproduction within this pathogen population and this should be considered in future disease control strategies, for instance, the consideration of longer potato crop rotations.

## Figures and Tables

**Table 1:** The origin of *Phytophthora infestans* isolates collected in 2010–2013 from different sites in Pskov region, north-west Russia, and results of mating type (A1 and A2) and metalaxyl resistance (R—resistant; I—intermediate; S—sensitive) testing.

Site	Sampling Year	Geographical Coordinates	Field Type *^Note^*	Number of Isolates				
				Mating Type		Response to Metalaxyl		
				A1	A2	R	I	S
Lakomtsevo (1)	2010	57.7528, 28.0898	SSC	7	0	0	1	5
Lakomtsevo (3)	2010	57.7531, 28.0899	SSC	1	3	0	0	3
Pechory	2010	57.8094, 27.8182	LSC	3	4	0	0	5
Styagly	2010	57.8395, 28.0865	SSC	4	4	0	0	4
Strugi Krasnye	2011	58.2654, 29.1143	SSC	4	5	0	0	9
Styagly (1)	2011	57.8405, 28.0864	SSC	4	1	0	0	5
Styagly (2)	2011	57.8403, 28.0866	SSC	4	0	0	0	4
Gusinets (1)	2012	57.7236, 27.8221	SSC	6	9	0	1	7
Gusinets (2)	2012	57.7239, 27.8210	SSC	5	10	0	1	6
Lezgi	2012	57.8121, 27.8245	LSC	1	0	0	0	1
Styagly (1)	2012	57.8395, 28.0863	SSC	7	12	0	3	12
Styagly (2)	2012	57.8401, 28.0866	SSC	16	4	0	0	12
Tyamsha	2012	57.7457, 28.1562	SSC	11	6	0	0	15
Zatrub’ye-Lebedy	2012	57.7591, 27.6860	LSC	14	8	0	9	8
Bereznyuk	2013	57.7799, 27.6879	LSC	4	3	2	2	3
Bulatnovo	2013	57.7907, 27.6582	LSC	5	2	2	0	5
Gusinets (1)	2013	57.7235, 27.8220	SSC	5	12	7	2	8
Gusinets (2)	2013	57.7234, 27.8203	SSC	5	9	4	1	7
Lezgi	2013	57.8124, 27.8294	LSC	7	9	7	1	6
Zatrub’ye-Lebedy	2013	57.7667, 27.6854	LSC	18	6	7	3	11
Total				131	107	29	24	136

*Note* LSC = large scale conventional; SSC = small scale conventional. The numbers in brackets indicate the number of field sites.

**Table 2:** Population genetic parameters for *P. infestans* over all sampling sites for 12 SSR loci.

SSR Locus	N_allele_	λ	*He*	E_5_	Pr.Exact
D13	7	0.255	0.257	0.391	0.000
G11	8	0.799	0.802	0.896	0.000
Pi02	4	0.462	0.464	0.585	0.100
Pi04	3	0.657	0.660	0.980	0.000
Pi4B	4	0.654	0.657	0.951	0.214
Pi63	4	0.633	0.636	0.906	0.561
Pi70	2	0.094	0.095	0.477	0.001
SSR2	2	0.493	0.494	0.986	0.840
SSR4	8	0.766	0.769	0.791	0.749
SSR6	2	0.437	0.439	0.887	1.000
SSR8	3	0.514	0.516	0.919	0.427
SSR11	3	0.611	0.613	0.897	0.582
Mean	4.17	0.532	0.534	0.806	

*Note* N_allele_ (allele number), Simpson’s index (λ) for gene diversity, expected heterozygosity (*He*), evenness (E_5_), clone corrected Hardy–Weinberg exact test of the alleles at each locus (Pr.exact).

**Table 3:** Analysis of molecular variance (AMOVA) of *P. infestans* isolates grouped into locations, years, and agricultural management practices (clone corrected).

Source	df	Sum of Squares	Mean Squares	Variance Components	Variation (%)	*p* Value
Among locations	9	33.245	3.694	0.142	5.306	0.207
Within locations	75	190.326	2.538	2.538	94.694	
Total	84	223.571	2.662	2.680		
Among years	3	11.846	3.949	0.069	2.568	0.226
Within years	79	206.762	2.617	2.617	97.432	
Total	82	218.608	2.666	2.686		
Among agricultural management practices	1	3.758	3.758	0.029	1.068	0.238
Within agricultural management practices	83	219.812	2.648	2.648	98.932	
Total	84	223.571	2.662	2.677		

*Note* degrees of freedom (df); significant at the *p* ≤ 0.05 level.

**Table 4:** Summary of the *P. infestans’* population diversity indices averaged over 12 loci.

Population	N	MLG	eMLG	λc	*He*	E_5_
Location						
Bereznyuk	7	6	6	0.952	0.451	0.937
Bulatnovo	7	5	5	0.905	0.486	0.931
Gusinets	34	17	4.73	0.829	0.516	0.479
Lakomtsevo	11	5	6	0.782	0.532	0.815
Lezgi	10	6	3	0.844	0.541	0.792
Pechory	6	3	6	0.733	0.234	0.898
Strugi Krasnye	9	6	8.48	0.889	0.492	0.866
Styagly	33	20	7	0.962	0.497	0.854
Tyamsha	7	7	7.31	1	0.505	1
Zatrub’ye-Lebedy	17	10	9.36	0.926	0.530	0.867
Mean value	14.1	8.5	6.29	0.882	0.478	0.843
Years						
2010	25	15	12.0	0.943	0.507	0.828
2011	18	11	11.0	0.928	0.521	0.841
2012	48	21	11.1	0.898	0.489	0.581
2013	50	36	15.9	0.985	0.551	0.887
Mean value	35.3	20.8	12.5	0.939	0.517	0.784
Agricultural management practices						
Large-scale conventional field (LSC)	47	30	30.0	0.978	0.532	0.871
Small-scale conventional field (SSC)	94	55	32.9	0.970	0.531	0.606
Mean value	70.5	42.5	31.5	0.974	0.532	0.739
Whole collection	141	85	36.8	0.984	0.533	0.663

*Note* N = number of individuals; MLG = number of multilocus genotypes observed; eMLG = expected number of MLGs at smallest size of at least ten; λc = Simpson’s index of MLG diversity corrected for sample size; He = Neil’s gene diversity; E5 = genotypic evenness. *Note*: 85 MLGs (2 MLGs were common between locations, as well as between agricultural management practices).

## Data Availability

Not applicable.

## Supplementary Materials

The following supporting information can be downloaded at: https://www.mdpi.com/article/10.3390/jof8050472/s1, Figure S1: Heatmap showing significant departures of locus from HWE for each location (a), year (b), and agricultural management practices (c), clone-corrected. This simple plot shows us loci in rows and populations in columns. Note that all loci shown in pink are loci suspected of not being in HWE with *p* ≤ 0.05. Be: Bereznyuk, Bu: Bulatnovo, Gu: Gusinets, La: Lakomtsevo, Le: Lezgi, Pe: Pechory, Stru: Strugi Krasnye, Sty: Styagly, Ty: Tyamsha, Za: Zatrub’ye-Lebedy. Figure S2: A bar plot of data showing the population membership probability assignments of *P. infestans* against their original populations for different locations (non-clone corrected). Bars of the same color represent the likelihood of the same genetic cluster based on analysis of the single sequence repeat marker data. Bars of mixed colors are admixed individuals/isolates. Figure S3: Discriminating alleles along axis 1 of discriminant analysis of principal components for locations based on analysis of the single sequence repeat marker data. Figure S4: A bar plot of data showing the population membership probability assignments of *P. infestans* against their original populations for different years (non-clone corrected). Bars of the same color represent the likelihood of the same genetic cluster based on analysis of the single sequence repeat marker data. Bars of mixed colors are admixed individuals/isolates. Figure S5: A bar plot of data showing the population membership probability assignments of *P. infestans* against their original populations for agricultural management practices (non-clone corrected). Bars of the same color represent the likelihood of the same genetic cluster based on analysis of the single sequence repeat marker data. Bars of mixed colors are admixed individuals/isolates. Figure S6: A discriminant analysis of the principal components (PC) scatterplot showing clustering of *P. infestans* isolates (clone corrected) according to (a) locations and (b) years, and density plot for (c) agricultural management practices. Scatterplots represent the distribution of individuals along the first two linear discriminants. Clusters are indicated by the color-coded ellipses for (a) and (b). Dots represent multilocus genotypes (in each population). The cross-validated number of the PCs used for the discriminant analysis is shown in the bar plots on the top left of the scatterplot. Indicated at the bottom right corner is the discriminant analysis eigenvalues, respectively. A total of 10, 23, and 9 cross-validated PCs explained 99.4%, 99.3%, and 98.1% of the variation for location, years, and agricultural management practices, respectively. Figure S7: Minimum spanning network (MSN) inferred from simple sequence repeat (SSR) markers of *P. infestans* (clone corrected) according to (a) locations, (b) years, and (c) agricultural management practices. Each node represents multilocus genotypes (MLGs) and the size is proportional to the number of individuals sharing the same SSR allele profile. The thickness of the lines represents the Bruvo’s genetic distance between two nodes (the further the genetic distance the lighter the color and thinner the line). Figure S8: A bar plot of clone corrected data showing the population membership probability assignments of *P. infestans* against their original populations for (a) locations, (b) years, and (c) agricultural management practices. Bars of the same color represent the likelihood of the same genetic cluster based on analysis of the single sequence repeat marker data. Bars of mixed colors are admixed individuals/isolates. Figure S9: Neighbor-net phylogenetic tree to access population pattern (clone corrected) on isolate collection at different (a) locations, (b) years, and (c) agricultural management practices. The clusters are color-coded. Table S1: Allele frequencies of SSR markers in 141 *P. infestans* isolates collected in Pskov region from 2010–2013. Table S2: Clone corrected standardized index of association (r¯d) within isolates of *P. infestans*, sampled at different locations, years, and agricultural management practices.
