# Supplementary material for: Highly Diverse Phytophthora infestans Populations Infecting Potato Crops in Pskov Region, North-West Russia
Source: J Fungi (Basel). 2022 Apr 30;8(5):472. doi: 10.3390/jof8050472 (PMC9147476; doi:10.3390/jof8050472)
Supplement: Supplementary file 1 [file jof-08-00472-s001.zip › jof-1640715-supplementary.pdf]

Supplementary materials

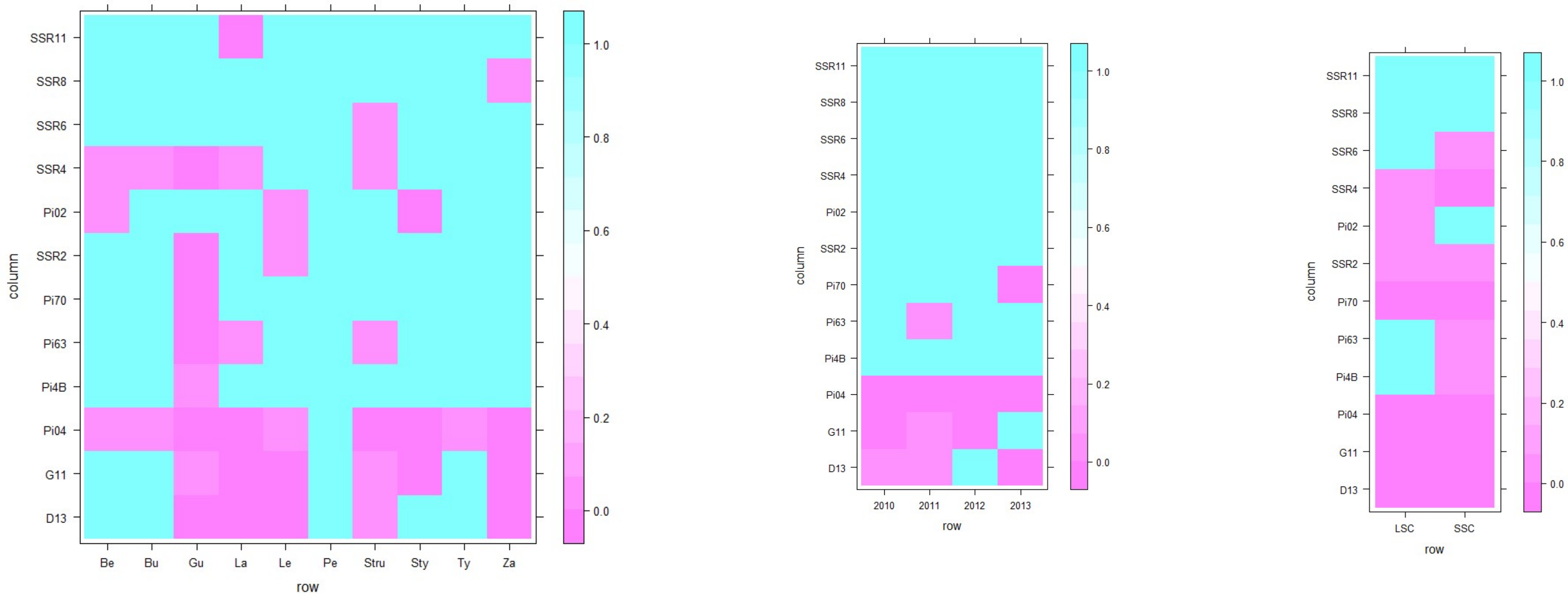

(a) (b) (c)

Figure S1. Heatmap showing significant departures of locus from HWE for each location (a), year (b) and agricultural management practices (c), clone-corrected. This simple plot shows us loci in rows and populations in columns. Note, that all loci shown in pink are loci suspected of not being in HWE with $P \leq 0.05$. Be: Bereznyuk, Bu: Bulatnovo, Gu: Gusinets, La: Lakomtsevo, Le: Lezgi, Pe: Pechory, Stru: Strugi Krasnye, Sty: Styagly, Ty: Tyamsha, Za: Zatrub'ye-Lebedy.

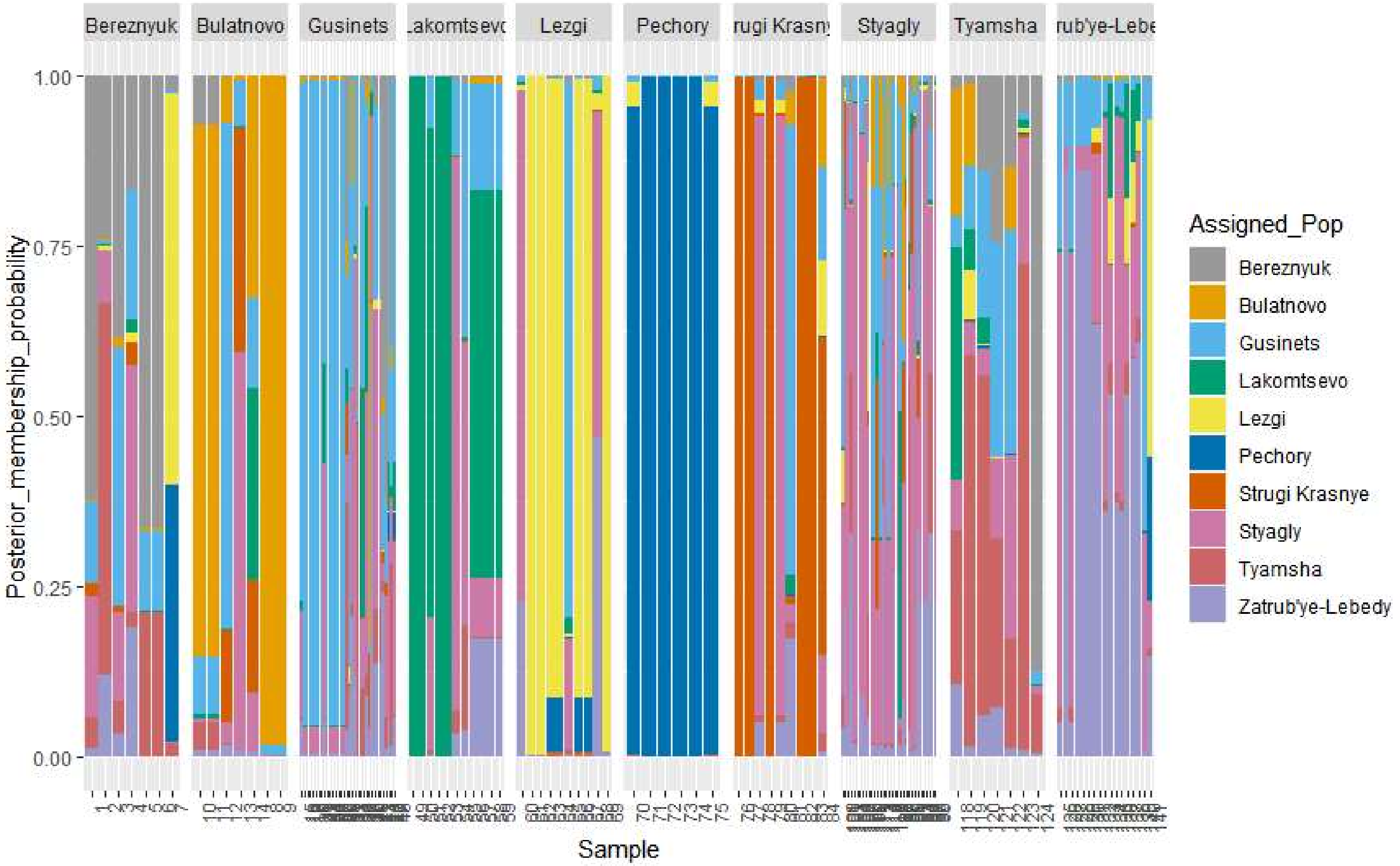

Figure S2. A bar plot of data showing the population membership probability assignments of *P. infestans* against their original populations for different locations (non-clone corrected). Bars of the same color represent the likelihood of the same genetic cluster based on analysis of the single sequence repeat marker data. Bars of mixed colors are admixed individuals/isolates.

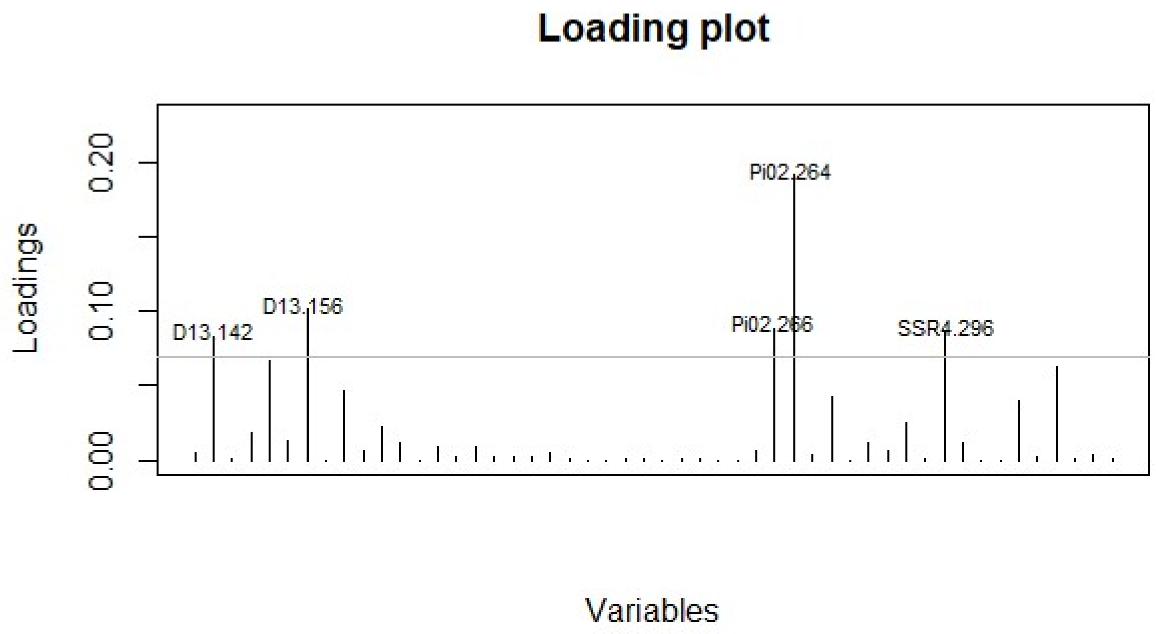

Figure S3. Discriminating alleles along axis 1 of discriminant analysis of principal components for locations based on analysis of the single sequence repeat marker data.

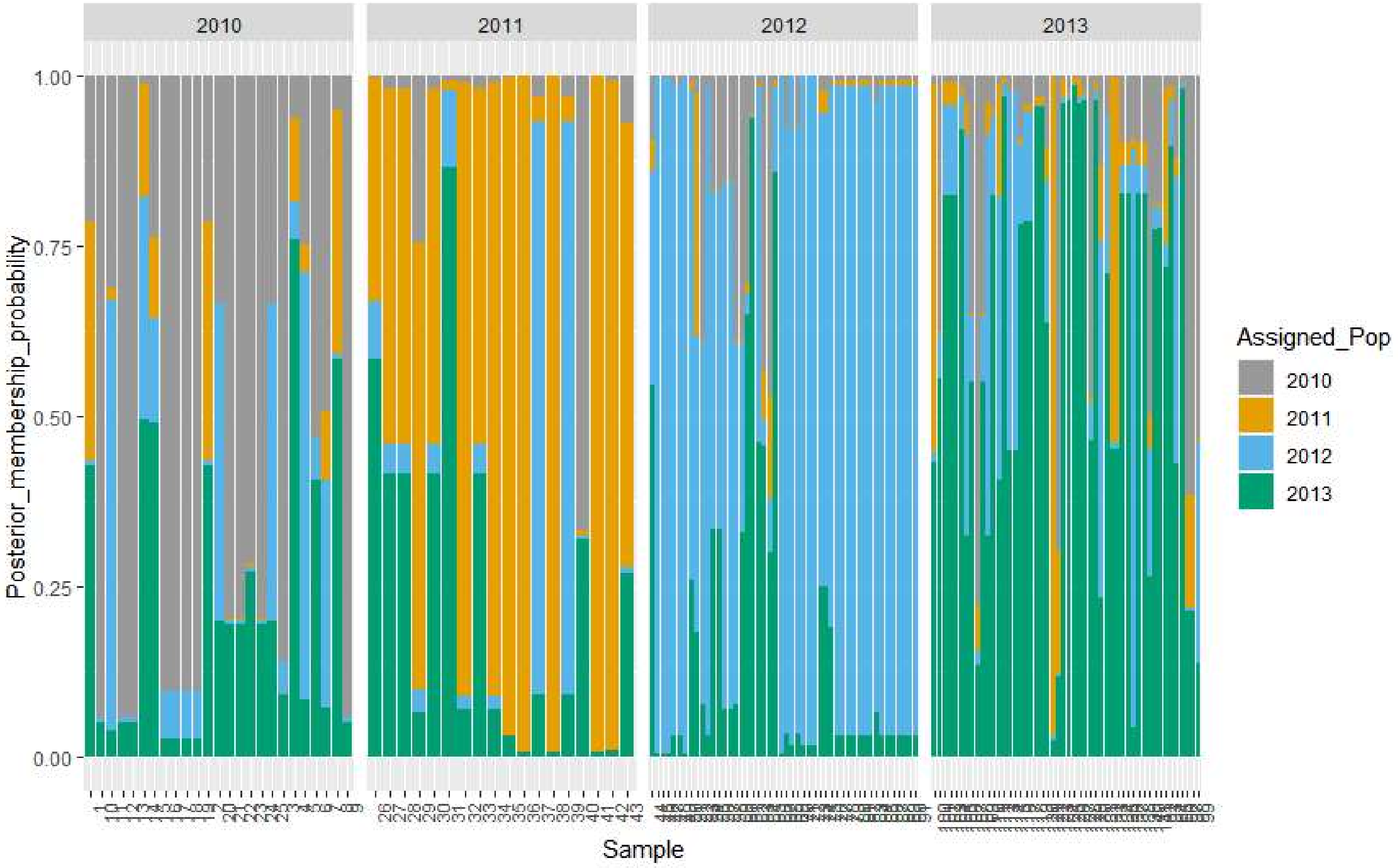

Figure S4. A bar plot of data showing the population membership probability assignments of *P. infestans* against their original populations for different years (non-clone corrected). Bars of the same color represent the likelihood of the same genetic cluster based on analysis of the single sequence repeat marker data. Bars of mixed colors are admixed individuals/isolates.

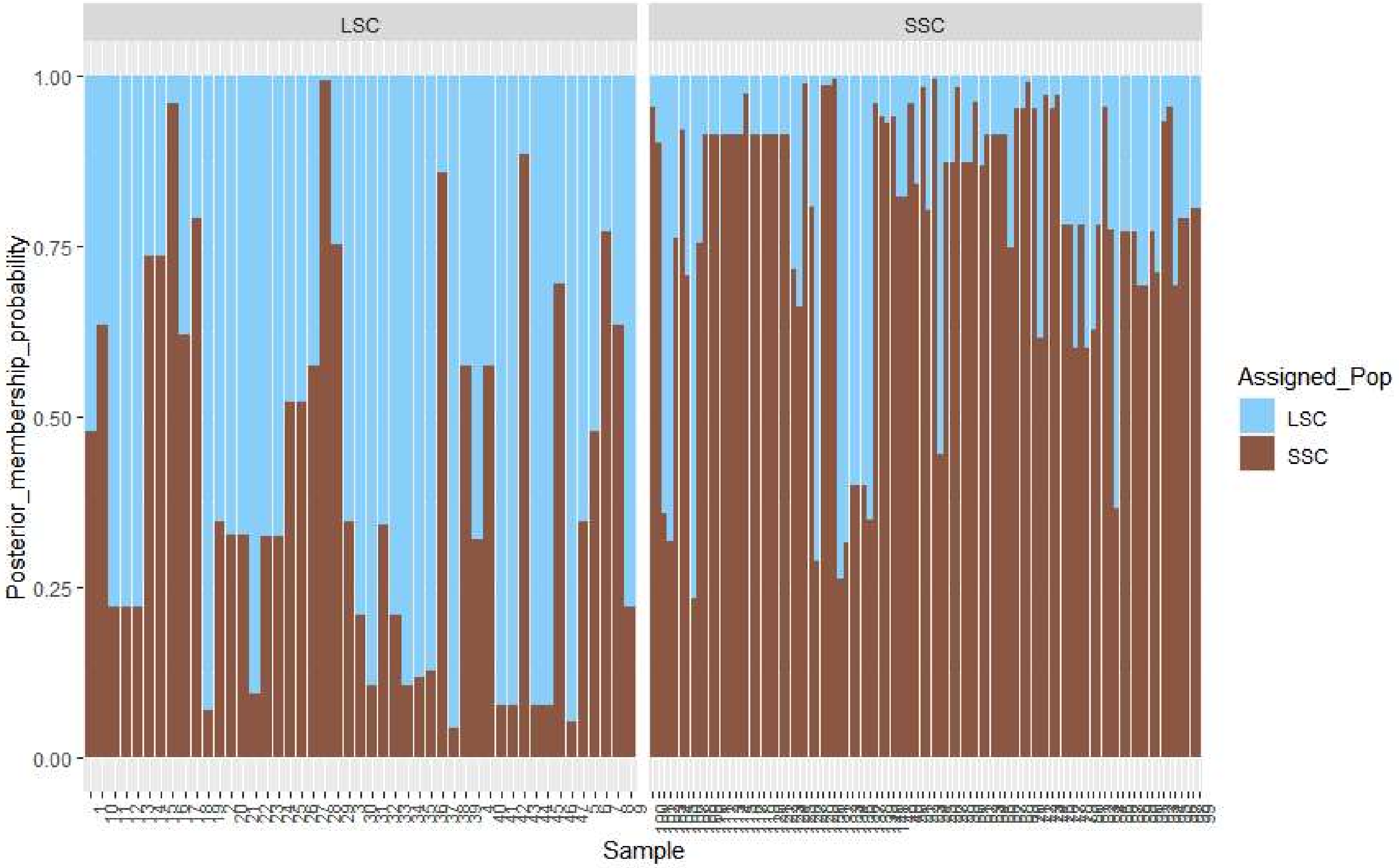

Figure S5. A bar plot of data showing the population membership probability assignments of *P. infestans* against their original populations for agricultural management practices (non-clone corrected). Bars of the same color represent the likelihood of the same genetic cluster based on analysis of the single sequence repeat marker data. Bars of mixed colors are admixed individuals/isolates.

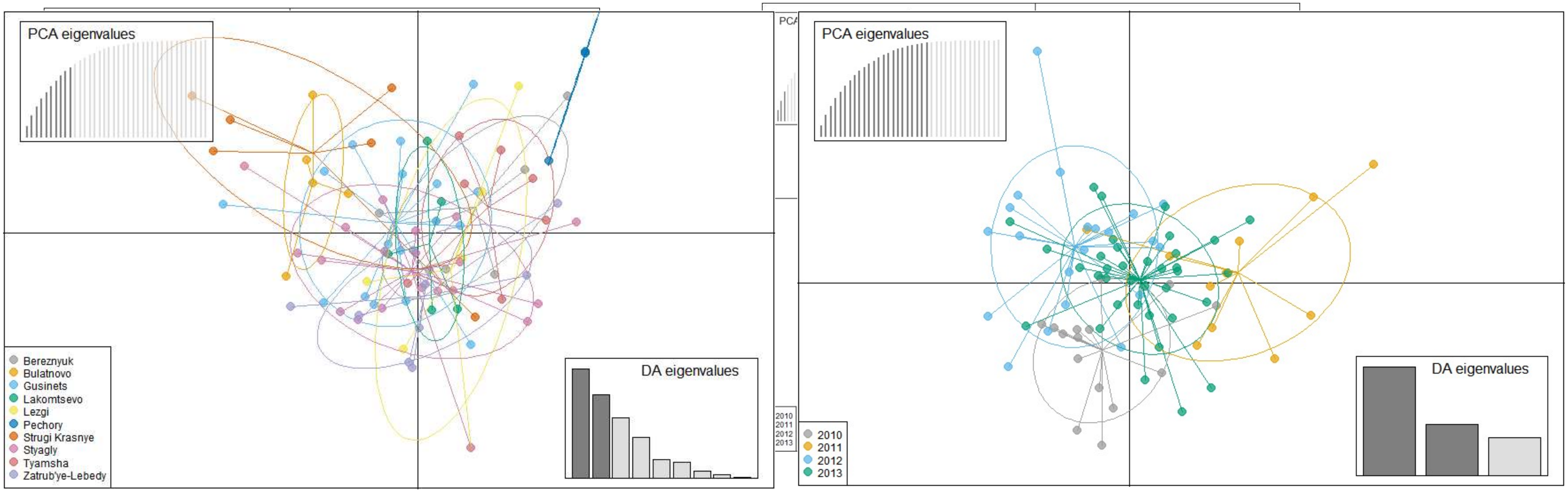

(a) **(b)**

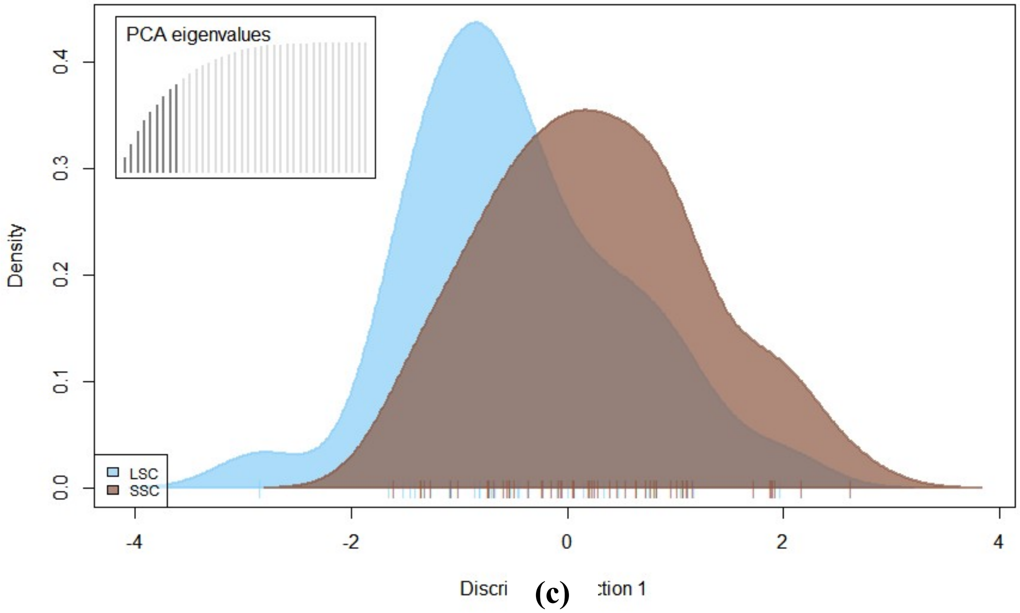

(c)

Figure S6. A discriminant analysis of the principal components (PC) scatterplot showing clustering of *P. infestans* isolates (clone corrected) according to (**a**) locations, (**b**) years, and density plot for (**c**) agricultural management practices. Scatterplots represent the distribution of individuals along the first two linear discriminants. Clusters are indicated by the color-coded ellipses for (a) and (b). Dots represent multilocus genotypes (in each population). The cross-validated number of the PCs used for the discriminant analysis is shown in the bar plots on the top left of the scatterplot. Indicated at the bottom right corner is the discriminant analysis eigenvalues, respectively. A total of 10, 23, and 9 cross-validated PCs explained 99.4%, 99.3%, and

98.1% of the variation for location, years and agricultural management practices, respectively.

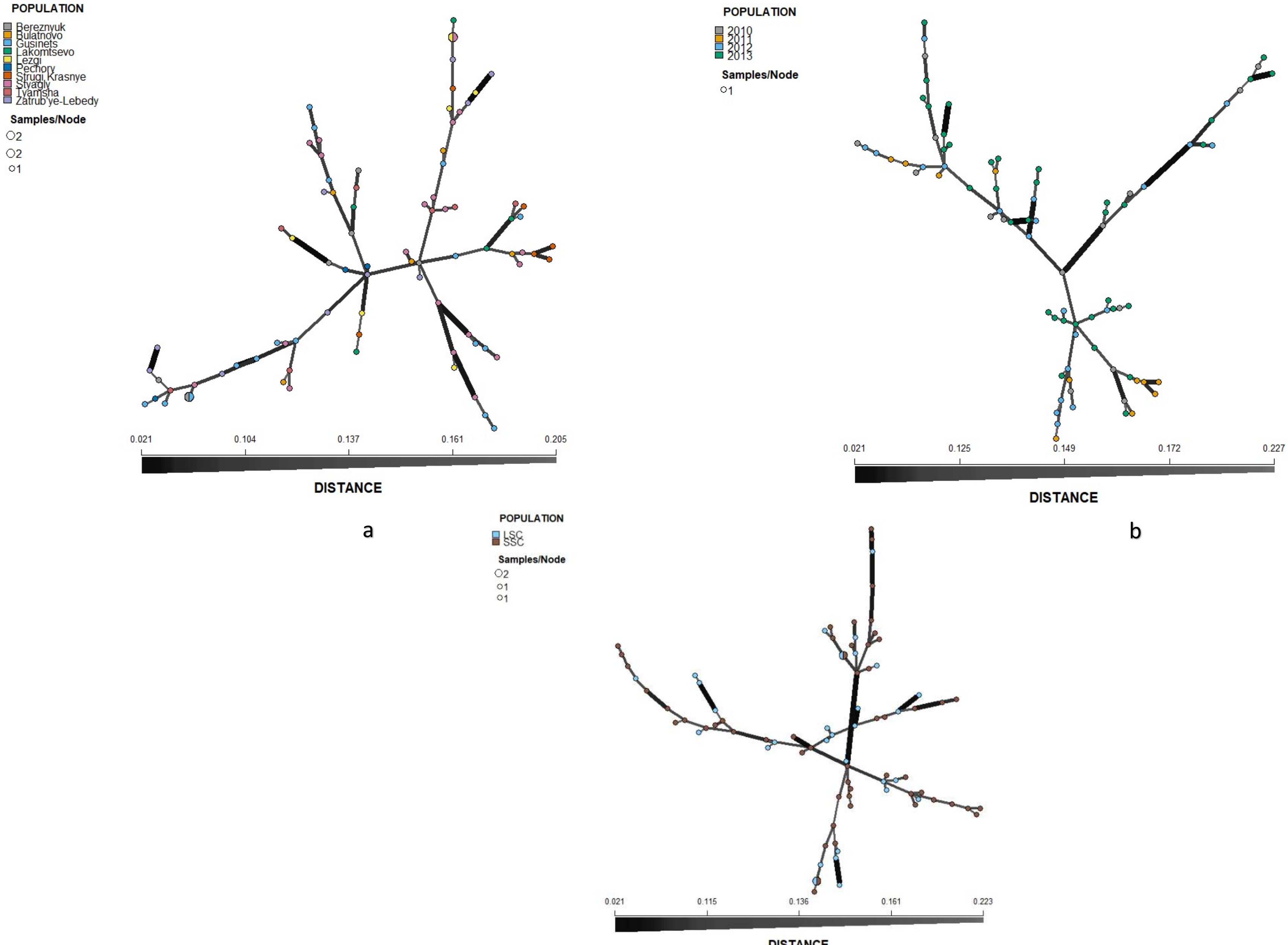

Figure S7. Minimum spanning network (MSN) inferred from simple sequence repeat (SSR) markers of *P. infestans* (clone corrected) according to (**a**) locations, (**b**) years, and (**c**) agricultural management practices. Each node represents multilocus genotypes (MLGs) and the size is proportional to the number of individuals sharing the same SSR allele profile. The thickness of the lines represents the Bruvo's genetic distance between two nodes (the further the genetic distance the lighter the color and thinner the line).

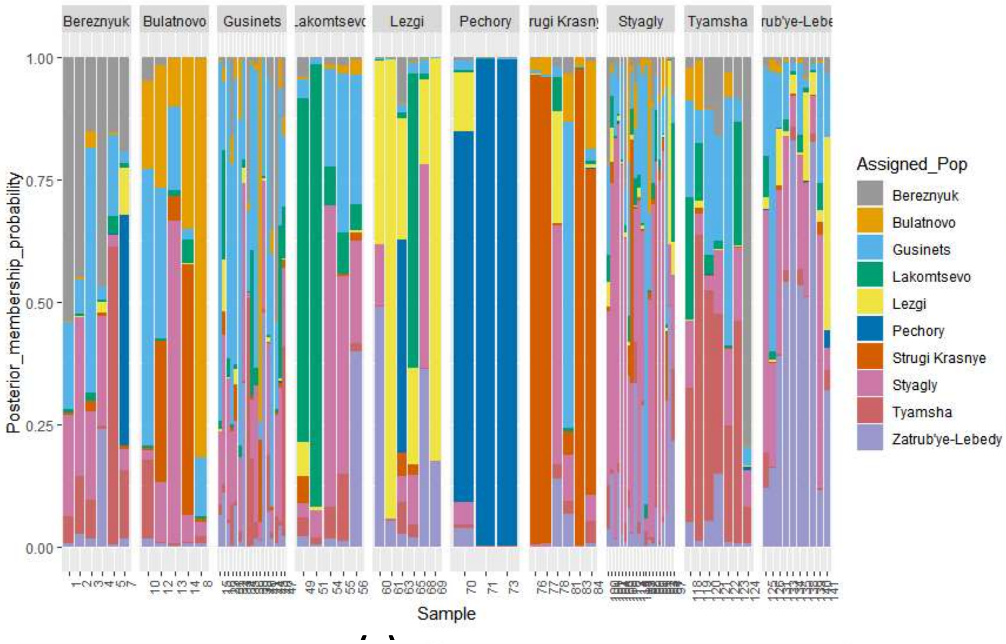

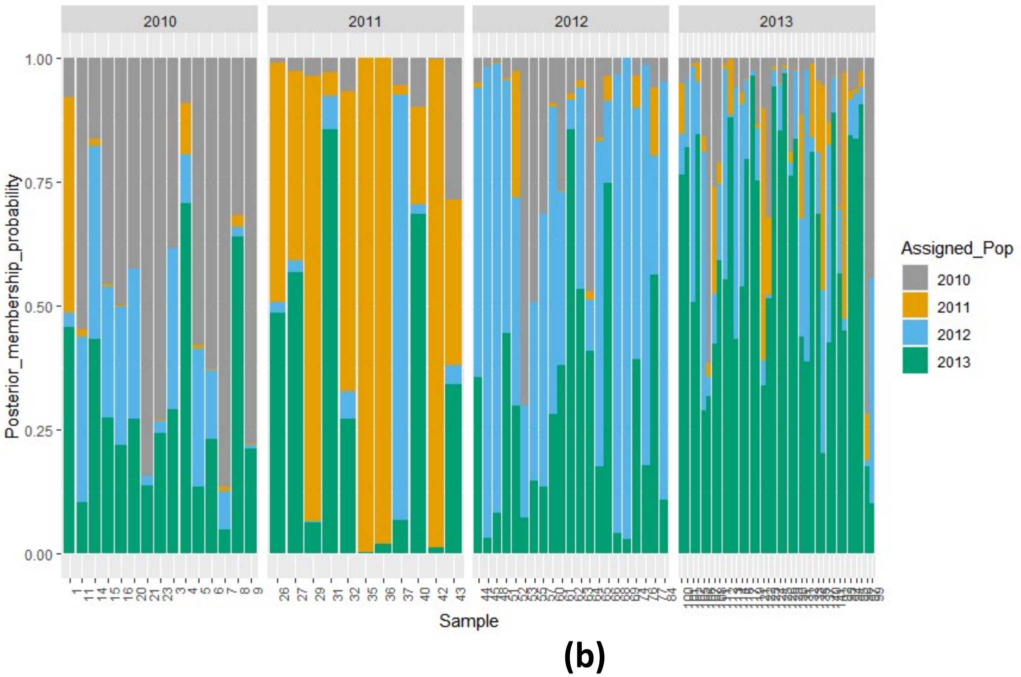

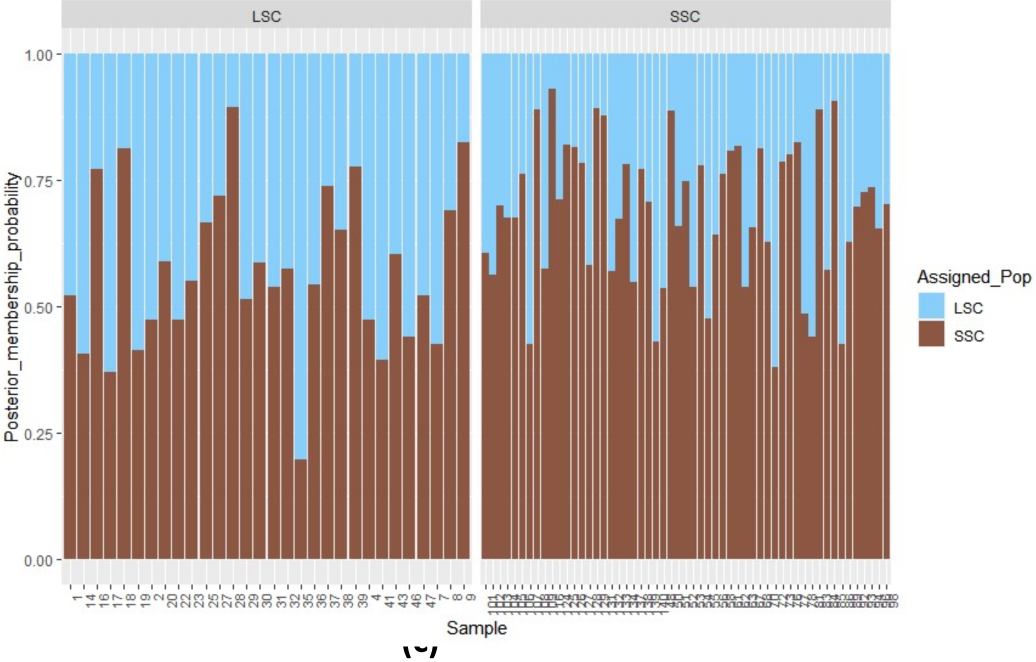

Figure S8. A bar plot of clone corrected data showing the population membership probability assignments of *P. infestans* against their original populations for (a) locations, (b) years, and (c) agricultural management practices. Bars of the same color represent the likelihood of the same genetic cluster based on analysis of the single sequence repeat

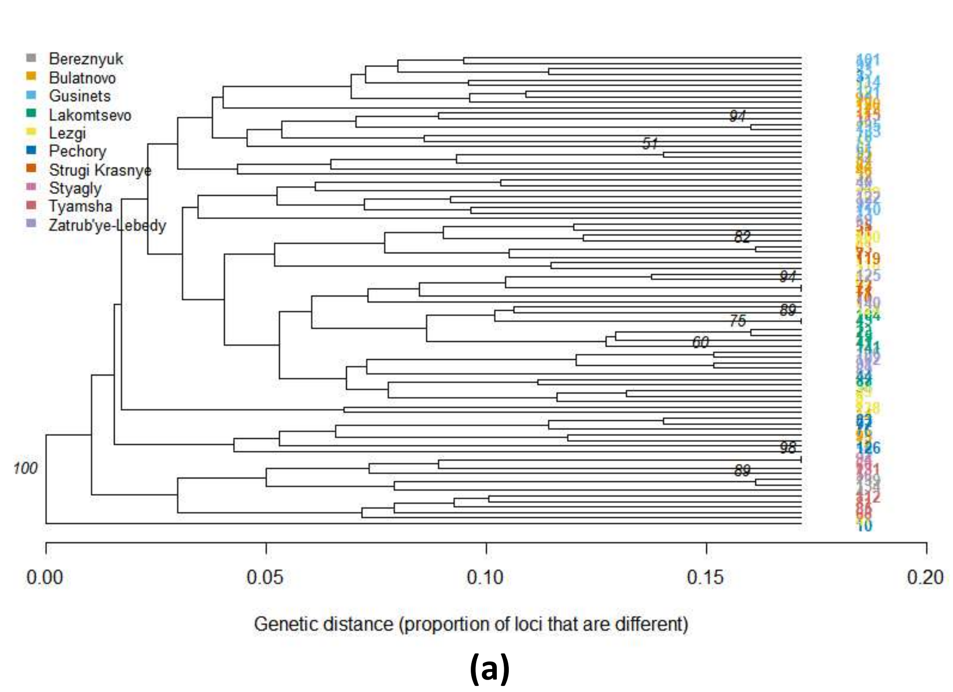

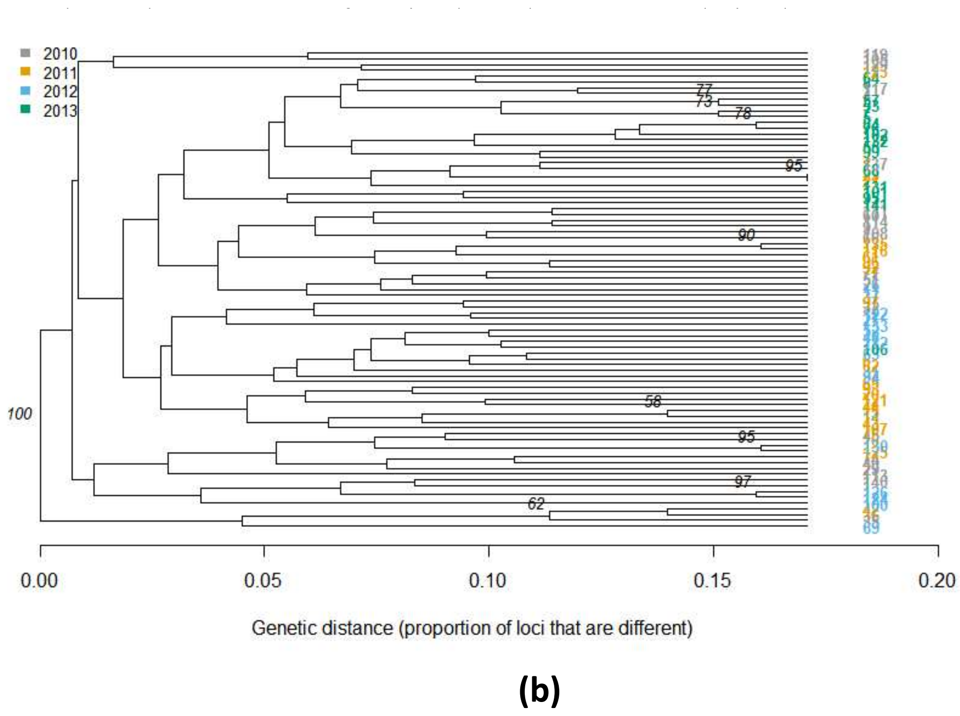

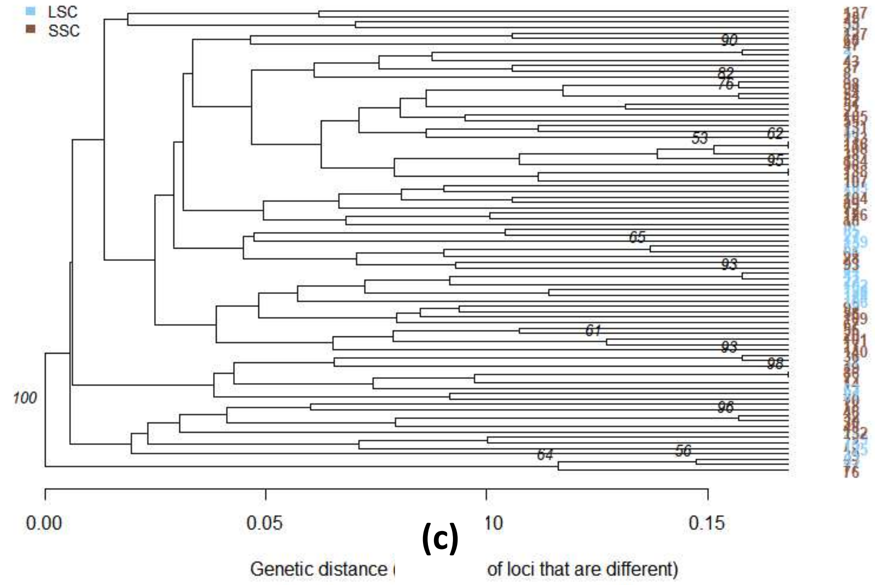

Figure S9. Neighbour-net phylogenetic tree to access population pattern (clone corrected) on isolate collection at different (a) locations, (b) years, and (c) agricultural management practices. The clusters are color-coded.

Table S1. Allele frequencies of SSR markers in 141 *P. infestans* isolates collected in Pskov region from 2010 – 2013.

SSR locus	Allele	Frequency	SSR locus	Allele	Frequency
Pi02	258	0.089	SSR4	284	0.376
	264	0.067		288	0.167
	266	0.131		290	0.028
	268	0.713		292	0.177
D13	132	0.016		294	0.167
	136	0.860		296	0.067
	138	0.022		298	0.011
	140	0.027		302	0.007
	142	0.054	SSR6	242	0.323
	156	0.011		244	0.677
	158	0.011	SSR8	260	0.528
G11	154	0.221		264	0.018
	156	0.237		266	0.454
	160	0.221	SSR11	331	0.170
	162	0.195		341	0.504
	166	0.011		355	0.326
	204	0.004	Pi63	270	0.248
	206	0.092		273	0.262
	210	0.019		276	0.004
Pi04	166	0.348		279	0.486

	168	0.259	Pi70	192	0.950
	170	0.394		195	0.050
Pi4B	205	0.404	SSR2	173	0.560
	213	0.238		175	0.440
	215	0.004			
	217	0.355			

Table S2. Clone corrected standardized index of association ($\bar{r}d$) within isolates of *P. infestans* sampled at different locations, years and agricultural management practices

**Locations**	$\bar{r}d$	*P*
Total	0.000	**0.456**
**Years**	$\bar{r}d$	*P*
2010	0.023	**0.083**
2011	0.026	**0.098**
2012	0.023	**0.056**
2013	0.002	**0.557**
Total	0.002	**0.625**
**Agricultural management practices**	$\bar{r}d$	*P*
LSC	0.003	**0.262**
SSC	0.004	**0.275**
Total	0.000	**0.500**

Bold *P* values ($P > 0.05$) indicate random mating is supported by the test. $\bar{r}d$, standardized index of association. Nb: an analysis was only performed for all locations together because of the low number of isolates in most of the locations.
